# Long-wavelength TCF-based fluorescence probes for the detection and intracellular imaging of biological thiols†
†Electronic supplementary information (ESI) available: All data supporting this study. See DOI: 10.1039/c8cc01661e


**DOI:** 10.1039/c8cc01661e

**Published:** 2018-04-17

**Authors:** Adam C. Sedgwick, Jordan E. Gardiner, Gyoungmi Kim, Maksims Yevglevskis, Matthew D. Lloyd, A. Toby A. Jenkins, Steven D. Bull, Juyoung Yoon, Tony D. James

**Affiliations:** a Department of Chemistry , University of Bath , Bath , BA2 7AY , UK . Email: t.d.james@bath.ac.uk ; Email: s.d.bull@bath.ac.uk; b Department of Chemistry and Nano Science , Ewha Womans University , Seoul 120-750 , Korea . Email: jyoon@ewha.ac.kr; c Drug & Target Development , Department of Pharmacy & Pharmacology , University of Bath , Claverton Down , Bath , BA2 7AY , UK

## Abstract

Two ‘turn on’ TCF-based fluorescence probes were developed for the detection of biological thiols (**TCF-GSH** and **TCFCl-GSH**). **TCF-GSH** was shown to have a high sensitivity towards glutathione (GSH) with a 0.28 μM limit of detection. Unfortunately, at higher GSH concentrations the fluorescence intensity of **TCF-GSH** decreased and toxicity was observed for **TCF-GSH** in live cells. However, **TCFCl-GSH** was shown to be able to detect GSH at biologically relevant concentrations with a 0.45 μM limit of detection. No toxicity was found for **TCFCl-GSH** and a clear ‘turn on’ with good photostability was observed for the exogenous addition of GSH, Cys and HCys. Furthermore, **TCFCl-GSH** was used to evaluate the effects of drug treatment on the levels of GSH in live cells.

## Abstract 2

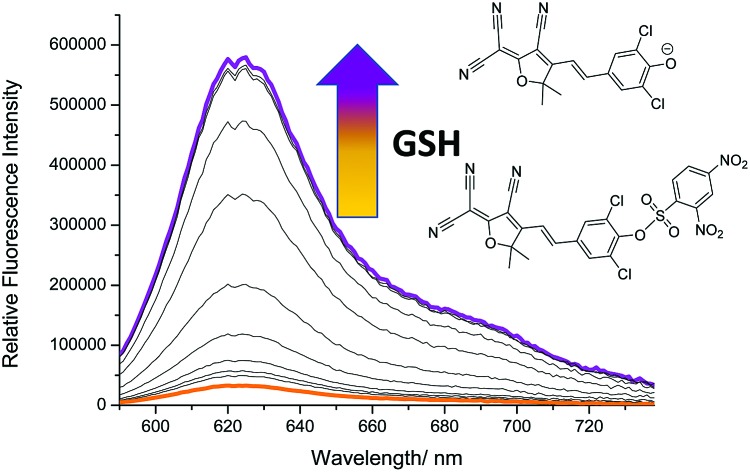
Two ‘turn on’ TCF-based fluorescence probes were developed for the detection of biological thiols (**TCF-GSH** and **TCFCl-GSH**).

## 

Glutathione (GSH), cysteine (Cys) and homocysteine (HCys) play a vital role in maintaining the biological redox homeostasis.^1^,^2^ GSH is a natural tripeptide (γ-l-glutamyl-l-cysteinyl-glycine), which exists in the thiol reduced form (GSH) and disulphide-oxidised (GSSG) form.^2^ GSH is the predominant form, which exists in millimolar concentrations in most cells where it functions as an antioxidant.^3^ Elevated levels of GSH are common in the presence of oxidative stress and the susceptibility of a cell towards reactive oxygen or nitrogen species (ROS/RNS) largely depends on the concentration of intracellular GSH.^4^–^7^ Therefore, the change in the level of GSH concentration has been associated with a number of diseases such as AIDS, liver damage, cancer and neurodegenerative disease (Alzheimer's disease).^6^,^7^ Interestingly, it was reported that at early stages of cell proliferation (S, G_2_ and M phases), GSH was found to localise at the nucleus. This was believed to prevent apoptosis and provide a reduced environment for transcription factors to bind to DNA.^8^

With our research, we are interested in the development of reaction based fluorescent probes for the detection of biologically relevant species to be used as powerful tools for the understanding of diseases.^9^–^13^ Currently, a number of fluorescent probes exist for the detection of biological thiols.^14^–^20^ However, long excitation/emission wavelength fluorescent probes are highly desirable as they allow deeper tissue penetration, minimal background auto-fluorescence from proteins and photodamage to the biological samples. Therefore, in this work we looked to develop TCF-based systems for the long wavelength detection of GSH.^11^

TCF-based fluorophores have an internal charge transfer (ICT) donor–π–acceptor (D–π–A) structure with long emission wavelengths (see ESI† – Scheme S1). As a result, TCF fluorophores have been used in many applications such as non-linear optic chromophores and fluorescent probes.^21^–^25^ Hilderbrand *et al.* previously developed a ‘turn on’ sulfonamide based TCF fluorescent probe for the detection of biological thiols.^26^ However, a PEG unit was required to provide aqueous solubility and cell permeability. The probe was successfully shown to detect biological thiols in 3T3 cells. We believed the synthesis of the analogous sulfonate ester would overcome the need for a PEG unit and provide a much simpler synthesis. The TCF fluorophore unit was synthesised as previously reported using the reaction of 3-hydroxy-3-methyl-2-butanone, malonitrile and NaOEt in EtOH. With the TCF unit in hand, the (D–π–A) systems **TCF-OH** and **TCFCl-OH** were isolated in high yield using microwave reaction conditions.^27^ The **TCF** phenols were then reacted with 2,4-dinitrobenzenesulfonylchloride to afford the desired fluorescent probes **TCF-GSH** and **TCFCl-GSH** in satisfactory yields (55% and 64%) (Fig. 1).

**Fig. 1:**
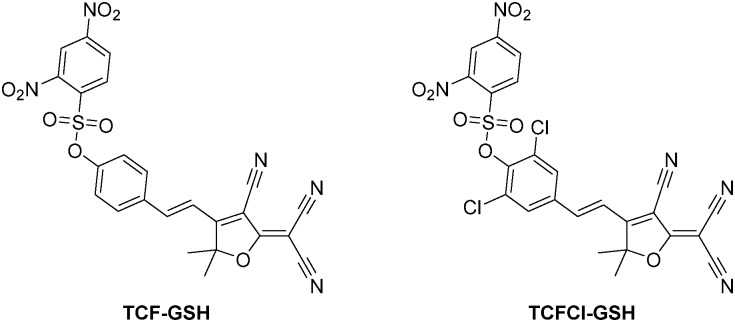
TCF-based fluorescent probes for the detection of biological thiols (**TCF-GSH** and **TCFCl-GSH**).

On the addition of GSH, both probes **TCF-GSH** and **TCFCl-GSH** change colour from yellow to purple (see ESI† – Fig. S1 and S2). We evaluated the fluorescence behaviour of **TCF-GSH**, in pH 8.0 buffer solution (20% v/v DMSO) (see ESI†–Fig. S3 and S4). Interestingly, 20% v/v DMSO was required for the reaction between the probe and the chosen biological thiol to take place. We then evaluated **TCF-GSH** for the detection of GSH, given that it is the most predominant biological thiol in cells. Remarkably, **TCF-GSH** was very sensitive towards GSH producing a full ‘turn on’ fluorescence response in the presence of 25 μM GSH. Unfortunately, at concentrations >50 μM the fluorescence intensity of **TCF-GSH** began to drop dramatically. This is due to attack of the TCF fluorophores by nucleophiles (Fig. 2)^28^ (see ESI† – Fig. S5–S11).

**Fig. 2:**
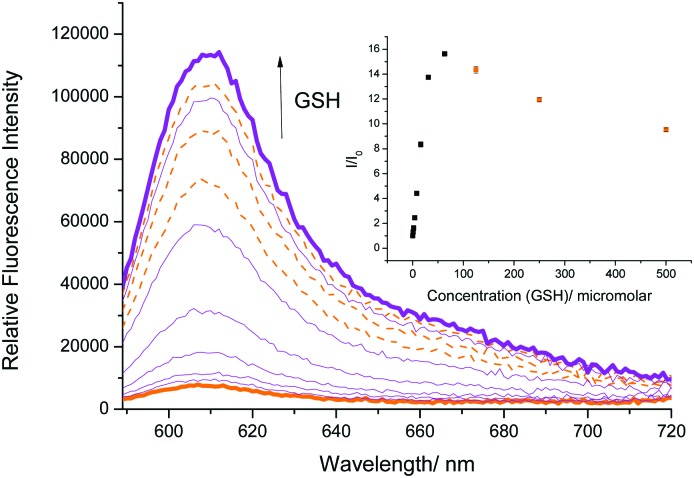
Fluorescence spectra of **TCF-GSH** (5 μM) with addition of GSH (0–500 μM) and 15 min wait between additions in PBS buffer solution, 20% v/v DMSO, pH 8.00 at 25 °C. *λ*_ex_ = 560 ± 15 nm. Orange dashed lines indicate fluorescence decrease at high GSH concentrations.

We then evaluated the selectivity of **TCF-GSH** towards other biologically relevant thiols and amino acids (see ESI† – Fig. S12 and S13). As predicted, **TCF-GSH** reacted with the other sulphydryl (R-SH) compounds, Cys and HCys with Cys producing the largest fluorescent response. However, the overall concentrations of both Cys and HCys are low in comparison to GSH in cells.^29^,^30^ **TCF-GSH** demonstrated an excellent selectivity for GSH against other amino acids. This excellent selectivity permitted the evaluation of **TCF-GSH** for the detection of exogenous and endogenously generated thiols in live cells. Sadly, despite **TCF-GSH** being sensitive towards GSH, we only observed a clear ‘off–on’ response for the exogenous addition of Cys in HeLa cells. Furthermore, **TCF-GSH** was shown to have toxicity in cell viability experiments (see ESI† – Fig. S16 and S17). **TCF-GSH** is therefore unsuitable for cell imaging experiments for the detection of biothiols. Interestingly, cellular imaging experiments did not require any additional additives to compensate for the 20% v/v DMSO required in the *in vitro* experiments.

We therefore turned our attention towards the fluorescence properties of **TCFCl-GSH**. In order to produce a fluorescence response, **TCFCl-GSH** also required pH 8.0 buffer solution (20% v/v DMSO). However, **TCFCl-GSH** was shown to be less sensitive towards the biological thiols and no decrease in fluorescence intensity was observed at higher concentrations (Fig. 3).

**Fig. 3:**
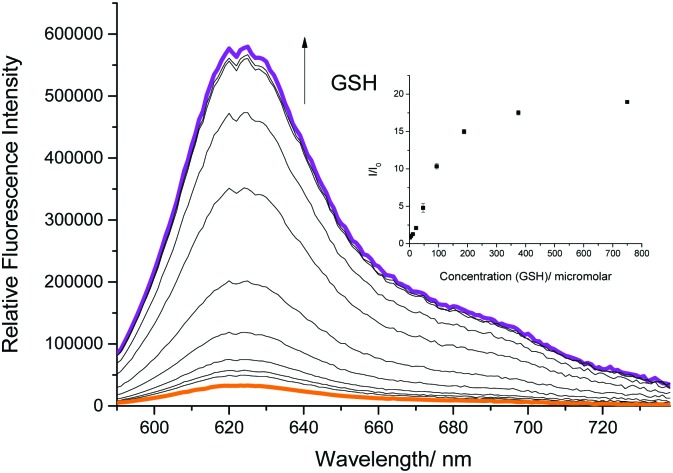
Fluorescence spectra of **TCFCl-GSH** (5 μM) with addition of GSH (0–750 μM) and 15 min wait between additions in PBS buffer solution, 20% v/v DMSO, pH 8.00 at 25 °C. *λ*_ex_ = 560 ± 15 nm.

We then evaluated the selectivity of **TCFCl-GSH** towards other biologically relevant thiols and amino acids (see ESI† – Fig. S14 and S15). As for **TCF-GSH**, **TCFCl-GSH** reacted with the R-SH containing amino acids Cys and HCys. While excellent selectivity for GSH was observed against other amino acids. This permitted the evaluation of **TCFCl-GSH** for the detection of exogenous and endogenous thiols in live cells. As shown in Fig. 4 (see ESI† – Fig. S18 and S19 for **TCF-GSH**), **TCFCl-GSH** displayed an already strong fluorescence response in live cells Fig. 4(a). This observation was due to the presence of endogenous thiols reacting with **TCFCl-GSH**. However, pre-treatment of HeLa cells with the thiol reactive *N*-methylmaleimide (NMM) led to the reduction of endogenous thiols and therefore low fluorescence intensity was observed when **TCFCl-GSH** was added Fig. 4(b). The addition of 200 μM of an exogenous thiol (Cys, HCys, or GSH-Methyl ester) led to a clear change in fluorescence intensity demonstrating the ability of **TCFCl-GSH** to detect thiols in cells.

**Fig. 4:**
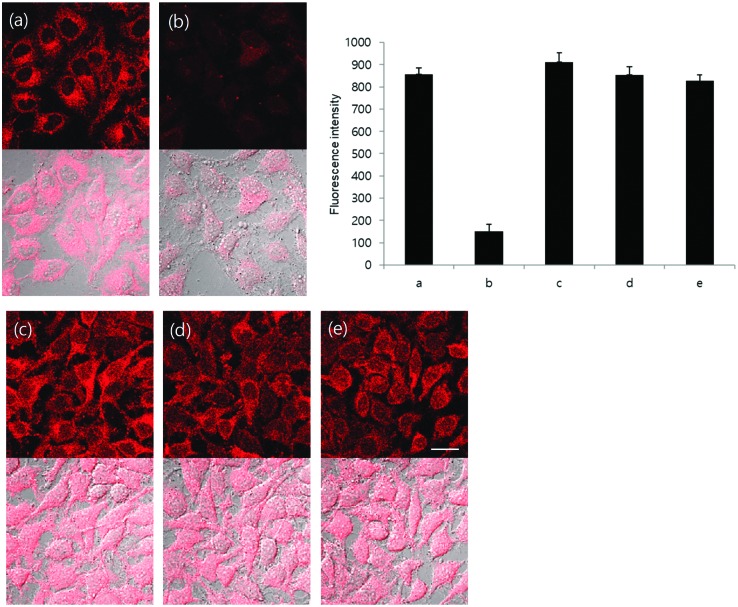
Fluorescence imaging in live cells. HeLa cells were preincubated with 0.2 mM NMM for 20 min and washed with Dulbecco's phosphate-buffered saline (DPBS) and incubated with 200 μM cysteine, homocysteine and GSH-MEE for 20 min. After washing with DPBS, cells were stained with 20 μM **TCFCl-GSH** for 20 min and fluorescence images acquired by confocal microscopy. (a) Only **TCFCl-GSH**, (b) NMM + **TCFCl-GSH**, (c) NMM + cysteine + **TCFCl-GSH**, (d) NMM + homocysteine + **TCFCl-GSH** and (e) NMM + GSH-MEE + **TCFCl-GSH**. Top: Fluorescence image (ex. 559 nm/em. 575–675 nm), bottom: merged image with DIC. Scale bar: 20 μm. Quantitative data of fluorescence intensity was calculated by FV10-ASW 4.0 software and measured per one cell. Results are expressed as mean ± standard deviation of three independent experiments.

We then evaluated the ability of **TCFCl-GSH** to detect changes in the concentration levels of endogenous thiols through the addition of drugs and reactive oxygen species (ROS) such as H_2_O_2_. It is well known that GSH protects against drug induced toxicity and acts as a ROS scavenger. Therefore in Fig. 5 and 6 (see ESI† – Fig. S20 and S21 for **TCF-GSH**), the addition of H_2_O_2_ (500 μM) or Cisplatin (200 μM) resulted in the depletion of the endogenous thiols and consequently reduced fluorescence was observed when **TCFCl-GSH** was added. Subsequently, the addition of the GSH producing drug *N*-acetylcysteine^31^ recovered the GSH levels resulting in a large increase in fluorescence intensity.

**Fig. 5:**
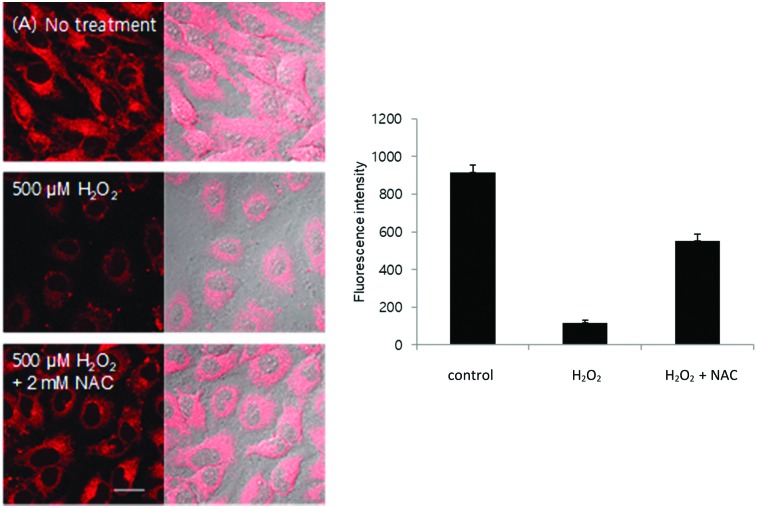
Intracellular fluorescence change caused by drug treatment. (A) HeLa cells were incubated with 500 μM H_2_O_2_ with or without 2 mM NAC for 6 h and stained with 20 μM **TCFCl-GSH** for 20 min. Quantitative data of fluorescence intensity was calculated by FV10-ASW 4.0 software and measured per one cell. Results are expressed as mean ± standard deviation of three independent experiments. Ex. 559 nm/em. 575–675 nm. Scale bar: 20 μm.

**Fig. 6:**
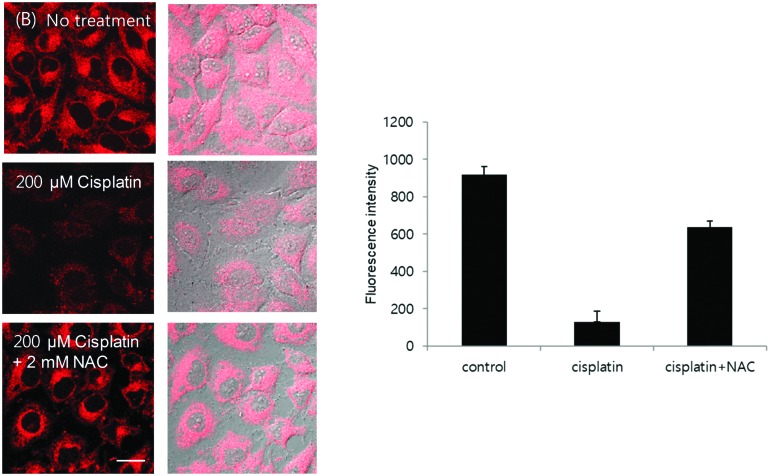
Intracellular fluorescence change caused by drug treatment. (B) HeLa cells were incubated with 200 μM cisplatin with or without 2 mM NAC for 6 h and stained with 20 μM **TCFCl-GSH** for 20 min. Quantitative data of fluorescence intensity was calculated by FV10-ASW 4.0 software and measured per one cell. Results are expressed as mean ± standard deviation of three independent experiments. Ex. 559 nm/em. 575–675 nm. Scale bar: 20 μm.

In summary, two ‘turn on’ TCF-based fluorescent probes have been developed for the detection of biological thiols (**TCF-GSH** and **TCFCl-GSH**). **TCF-GSH** was shown to have a high sensitivity towards glutathione (GSH). Unfortunately, at higher GSH concentrations the fluorescence intensity of **TCF-GSH** decreased and toxicity was observed in live cells making it unsuitable for cellular imaging. However, **TCFCl-GSH** was shown to be able to detect GSH at biological relevant concentrations. Also, no toxicity was observed for **TCFCl-GSH** and a clear ‘turn on’ response was observed upon the exogenous addition of GSH, Cys and HCys. Furthermore, **TCFCl-GSH** was able to evaluate the effects of drug treatment and the addition of ROS (H_2_O_2_) on live cells, both of which resulted in a depletion of cellular GSH levels and a reduced fluorescence intensity. Subsequent, addition of NAC increased the GSH levels and enhanced the observed fluorescence intensity.

We would like to thank the EPSRC, the University of Bath and Prostate Cancer UK (PG14-009) for funding. ACS and JEG thank the EPSRC for studentships. TDJ wishes to thank the Royal Society for a Wolfson Research Merit Award. NMR characterisation facilities were provided through the Chemical Characterisation and Analysis Facility (CCAF) at the University of Bath (www.bath.ac.uk/ccaf). The EPSRC UK National Mass Spectrometry Facility at Swansea University is thanked for analyses. JY thanks the support from the National Research Foundation of Korea (NRF), which was funded by the Korea government (MSIP) (No. 2012R1A3A2048814). ACS, MY, MDL and TDJ are members of the Cancer Research@Bath (CR@B) network.

## Conflicts of interest

No conflicts of interest.

## Supplementary Material

Supplementary information
