# Supplementary material for: Long-wavelength TCF-based fluorescence probes for the detection and intracellular imaging of biological thiols
Source: Chem Commun (Camb). 2018 Apr 17;54(38):4786–9. doi: 10.1039/c8cc01661e (PMC5944426; doi:10.1039/c8cc01661e)
Supplement: Supplementary file 1 [file CC-054-C8CC01661E-s001.pdf]

Electronic Supplementary Material (ESI) for ChemComm.
This journal is © The Royal Society of Chemistry 2018

Supplementary Information

Long-wavelength fluorescent TCF-based probes for the detection and intracellular imaging of biological thiols

Adam C. Sedgwick[a]*, Jordan E. Gardiner[a], Gyoungmi Kim[b], Maksims Yevglevskis[c], Matthew D. Lloyd[c], Toby A. Jenkins[a], Steven D. Bull[a]*, Juyoung Yoon[b]* and Tony D. James[a]*

[a]Department of Chemistry, University of Bath, Bath, BA2 7AY, U.K.

[b]Department of Chemistry and Nano science, Ewha Womans University, Seoul 120-750, Korea.

[c]Drug & Target Development, Department of Pharmacy & Pharmacology, University of Bath, Claverton Down, Bath, BA2 7AY, UK.

Email: T.D.James@bath.ac.uk, S.D.Bull@bath.ac.uk, jyoon@ewha.ac.kr

Content

1. **Reaction mechanism**
2. **UV analysis**
3. **Stability of the fluorophores towards GSH**
4. **Fluorescence analysis**
5. **Cell viability**
6. **Cell imaging**
7. **Photobleaching analysis**
8. **Quantum yield and Limit of detection**
9. **Experimental**
10. **NMR**
11. **References**

1. Reaction mechanism

As previously reported[1,2] the system has an internal charge transfer (ICT) donor-π-acceptor (D-π-A) structure. Before the addition of GSH, it is a pull-pull system. The addition of GSH leads to the deprotection of the phenol by reacting with the dinitrobenzenesulfonyl group. This produces the push-pull ICT system with a colorimetric and fluorescence response.

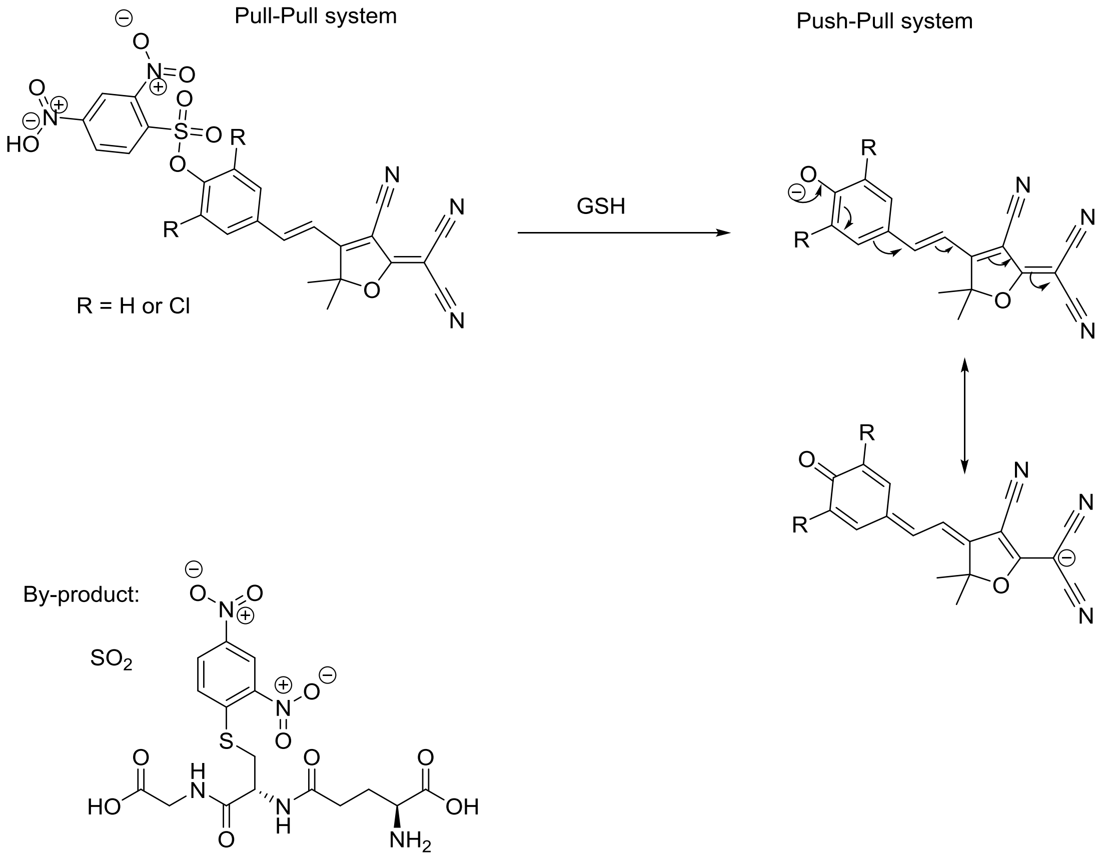

Scheme 1. Reactivity of the TCF-Based fluorescent probes towards GSH

2. UV analysis

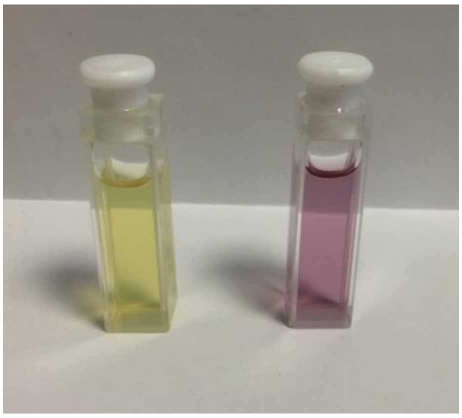

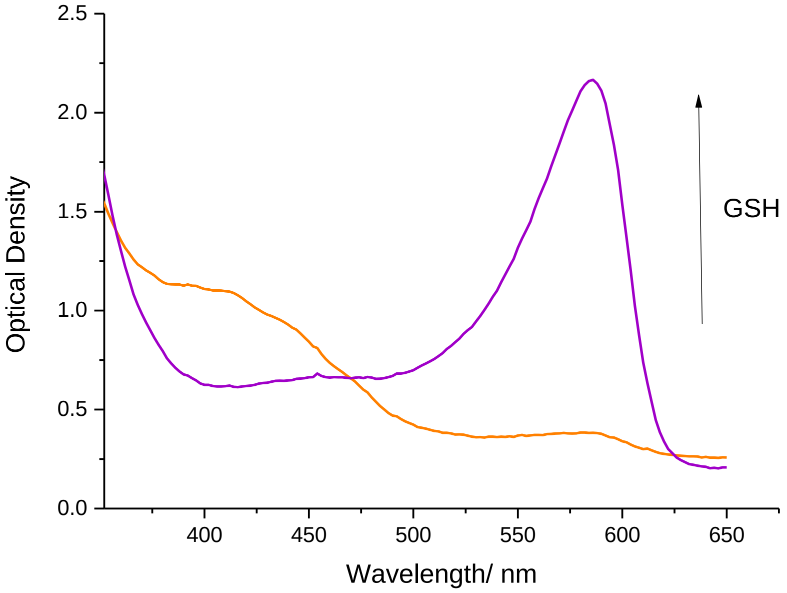

Fig S1. UV Spectra of **TCF-GSH** (10 µM) before (black) and after (purple) addition of GSH (50 µM) in PBS buffer solution, 20 % v/v DMSO, pH 8.00 at 25 °C. Wait 30 mins before measurement

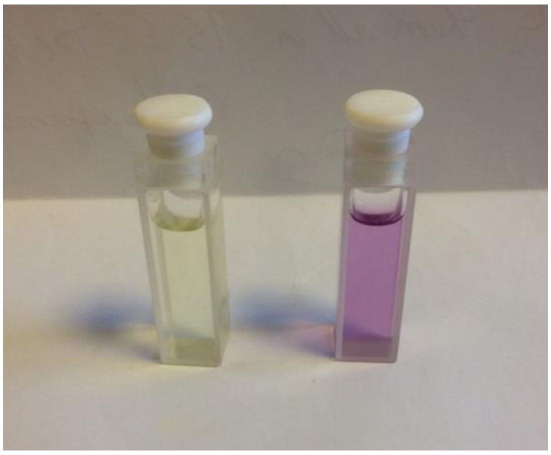

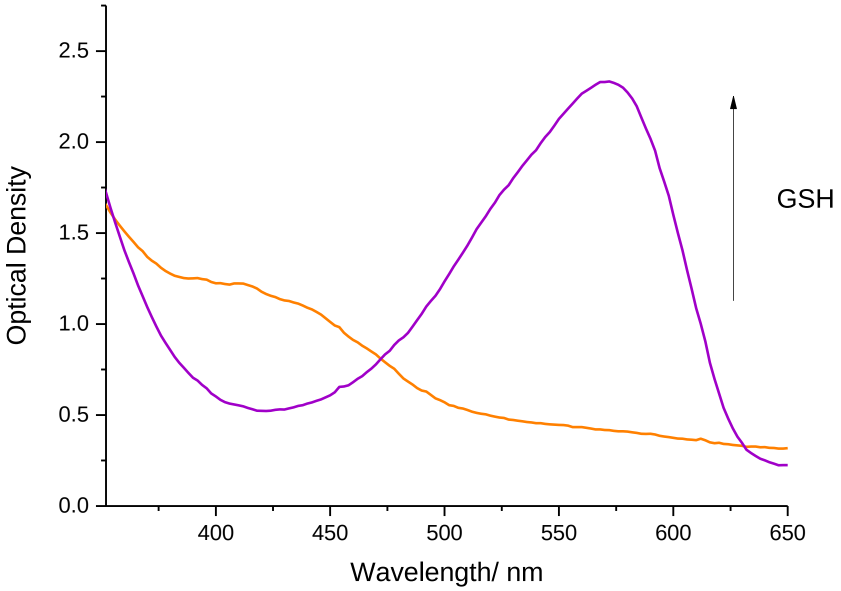

Fig S2. UV Spectra of **TCFCl-GSH** (10 μM) before (orange) and after (purple) addition of GSH (200 μM) in PBS buffer solution, 20 % v/v DMSO, pH 8.00 at 25 ^{o}C. Wait 30 mins before measurement

3. Fluorescence analysis

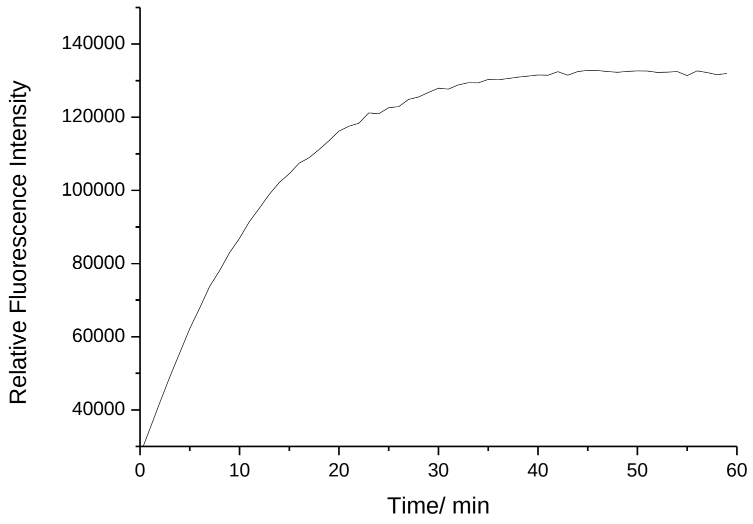

Fig. S3. Fluorescence change of **TCF-GSH** (5 μM) against time with the addition of GSH (100 μM) in PBS buffer solution, 20 % v/v DMSO, pH 8.00 at 25 °C. λex = 560 ± 15 nm/ λem = 610 ± 20 nm

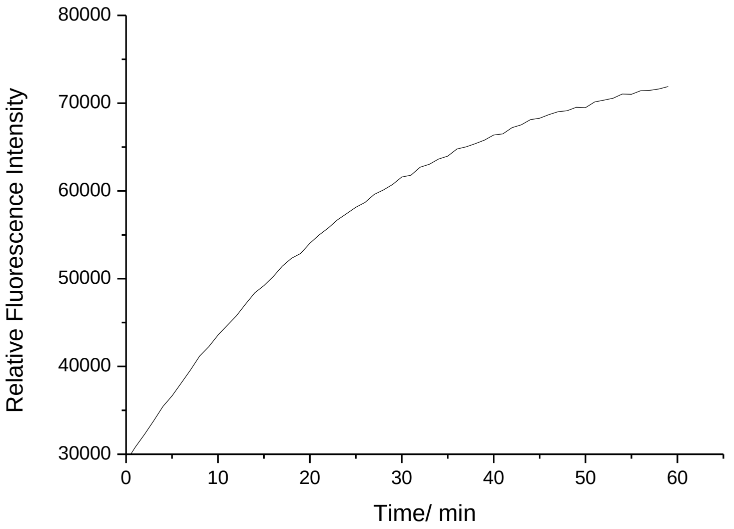

Fig S4. Fluorescence change of **TCFCl-GSH** (5 μM) against time with the addition of GSH (100 μM) in PBS buffer solution, 20 % v/v DMSO, pH 8.00 at 25 °C. λex = 560 ± 15 nm/ λem = 610 ± 20 nm

4. Stability of TCF fluorophores towards GSH

As shown in the manuscript (Fig. 2), at higher GSH concentrations the fluorescence intensity began to decrease. Therefore, we investigated the stability of the TCF phenols towards the addition of GSH. As illustrated below in Fig. S11, there is a clear colour change from purple to brown with the addition of 2 mM GSH. A clear decrease in fluorescence intensity was observed with the addition of GSH (Fig. S4)

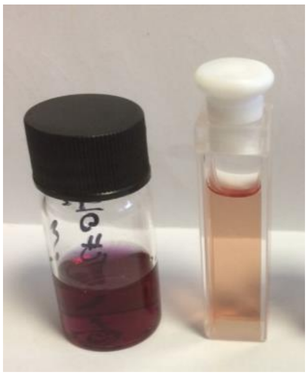

Fig S5. Vial on left – **TCF-OH – no GSH.** Cuvette on the right **TCF-OH – with GSH**

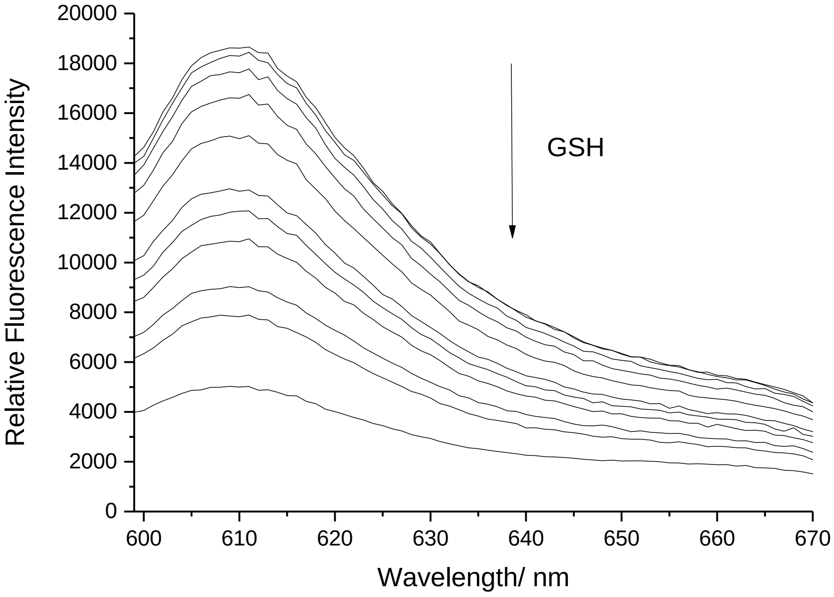

Fig S6. Fluorescence spectra of **TCF-OH** (5 µM) with the addition of GSH, (0 – 400 µM) in PBS buffer solution, 20 % v/v DMSO, pH 8.00 at 25 ºC. λex = 560 ± 15 nm

Interestingly, only a slight colour change was observed for **TCFCl-OH** with the addition of 2 mM GSH (Fig. S13).

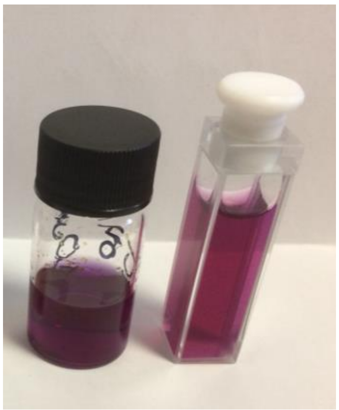

Fig S7. Vial on left – **TCFCl-OH – no GSH.** Cuvette on the right **TCFCl-OH – with GSH**

Despite only a small change in colour, a decrease in fluorescence intensity with the addition of GSH to **TCFCl-OH** – Fig. S14. However, in comparison to TCF-OH, there was smaller change in the fluorescence intensity indicating a greater stability to GSH.

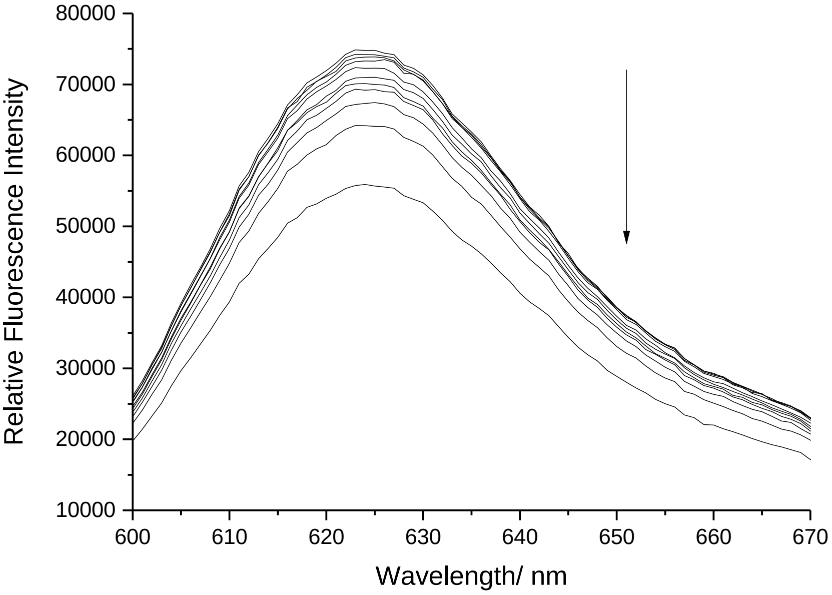

Fig S8. Fluorescence spectra of **TCFCl-OH** (5 µM) with the addition of GSH (0 – 400 µM) in PBS buffer solution, 20 % v/v DMSO, pH 8.00 at 25 °C. λex = 560 ± 15 nm

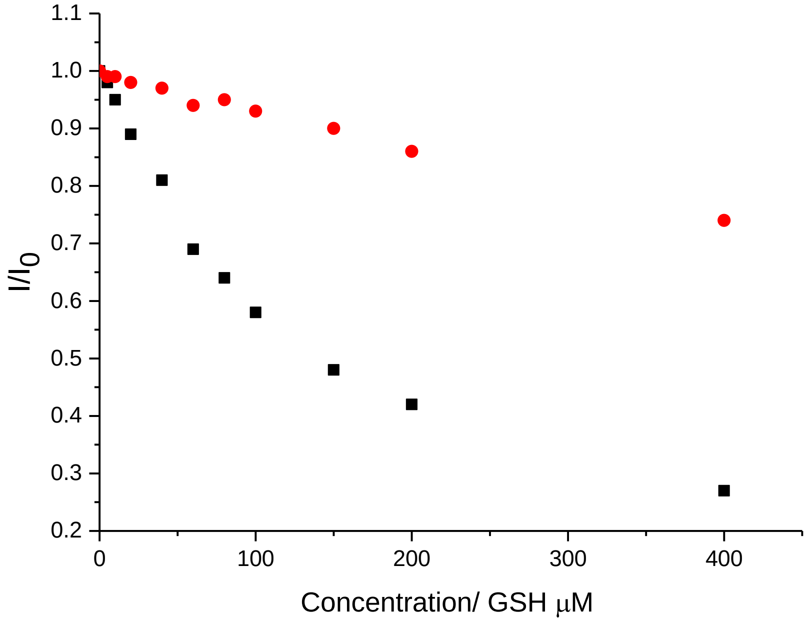

Fig. S9. Fluorescence intensity changes of **TCF-OH** ● (5 μM) and **TCFCl-OH** ■ (5 μM) with the addition of GSH (0 – 400 μM) in PBS buffer solution, 20 % v/v DMSO, pH 8.00 at 25 ^{o}C. λex = 560 ± 15 nm/ λem = 615 (TCF-OH)/ 625 (TCFCl-OH) nm

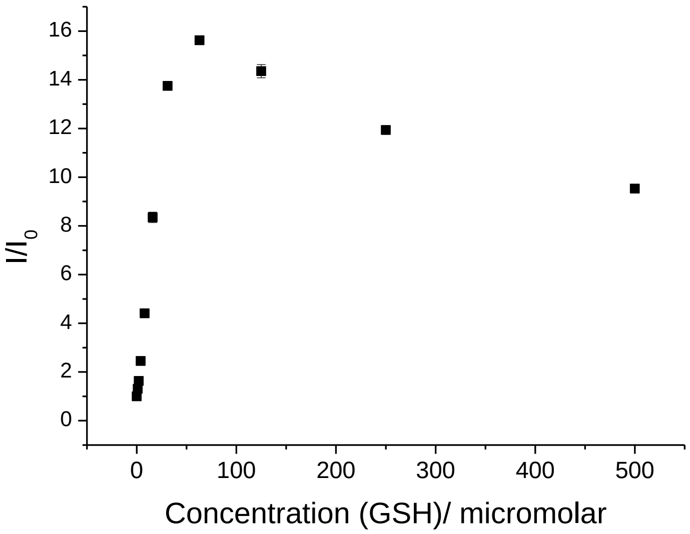

Fig S10. Fluorescence intensity change of **TCF-GSH** (5 µM) with the addition of GSH (0 – 500 µM) in PBS buffer solution, 20 % v/v DMSO, pH 8.00 at 25 ºC. λex = 560 ± 15 nm/ λem = 615 nm

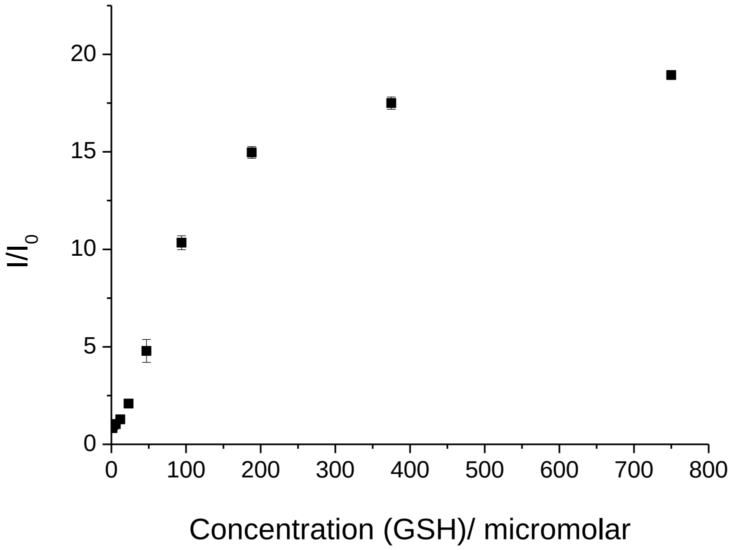

Fig S11. Fluorescence intensitys change of **TCFCl-GSH** (5 µM) with the addition of GSH (0 – 750 µM) in PBS buffer solution, 20 % v/v DMSO, pH 8.00 at 25 ºC. λex = 560 ± 15 nm/ λem = 625 nm

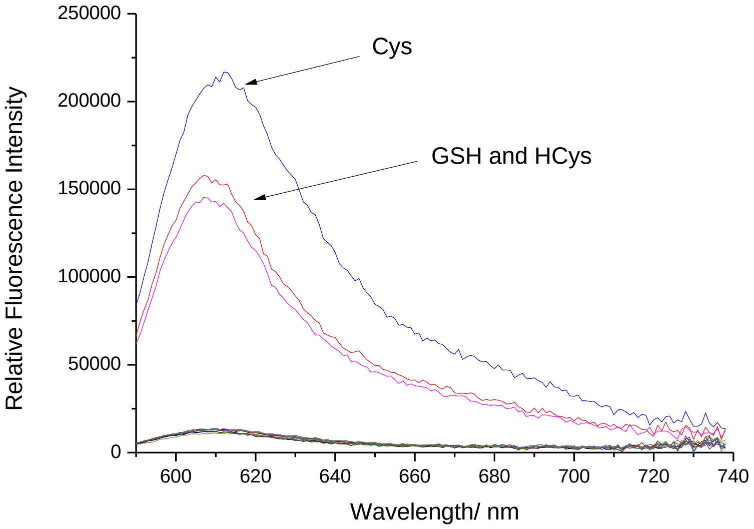

Fig S12. Fluorescence spectra of **TCF-GSH** (5 µM) with the addition of GSH, Cys, HCys and various other amino acids in PBS buffer solution, 20 % v/v DMSO, pH 8.00 at 25 °C. λex = 560 ± 15 nm

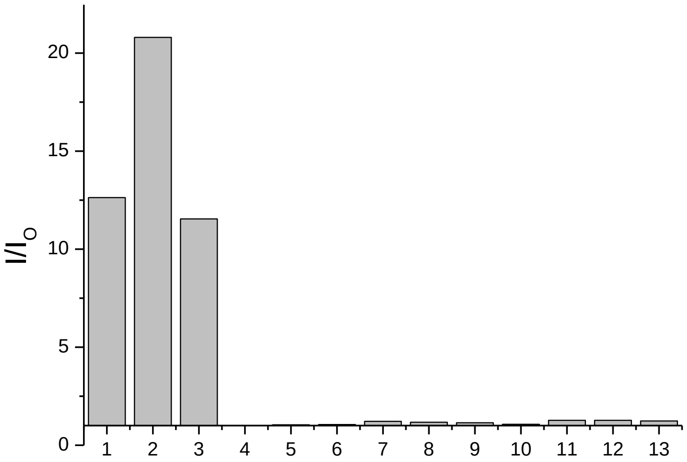

Fig S13. Selectivity bar chart of **TCF-GSH** (5 µM) with the addition of 100 µM GSH, Cys, HCys and various other amino acids (1 – GSH, 2 – Cys, 3 – Hcys, 4 – control, 5 – proline, 6 – glutamic acid, 7 – serine, 8 – Lysine, 9 – arginine, 10 – aspartic acid, 11 – Phenylalanine, 12 – valine, 13 – isoleucine) in PBS buffer solution, 20 % v/v DMSO, pH 8.00 at 25 °C. λex = 560 ± 15 nm/ λem = 625 nm

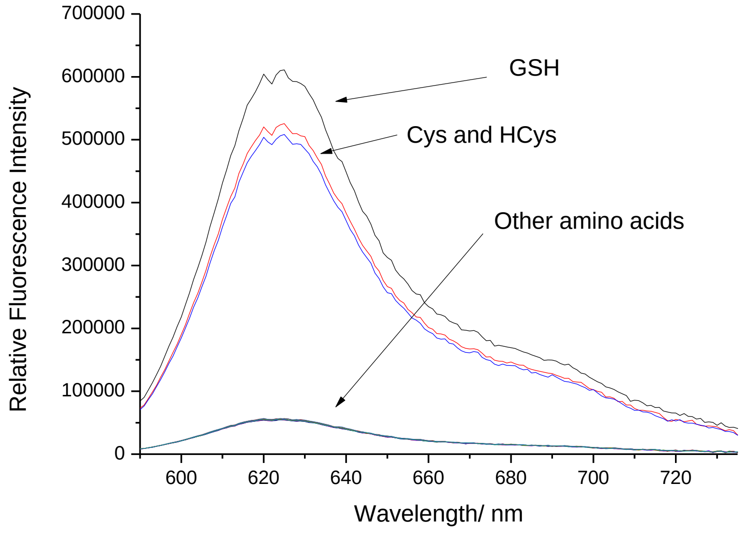

Fig S14. Fluorescence spectra of **TCFCl-GSH** (5 µM) with the addition of GSH, Cys, HCys and various other amino acids in PBS buffer solution, 20 % v/v DMSO, pH 8.00 at 25 ºC. λex = 560 ± 15 nm

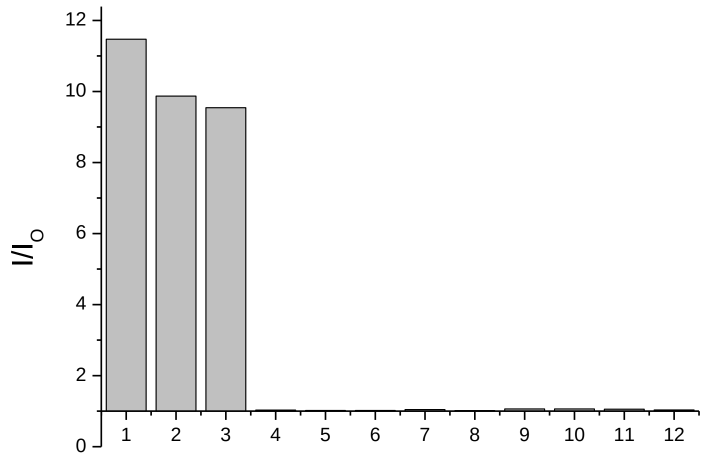

Fig S15. Selectivity bar chart of **TCFCl-GSH** (5 µM) with the addition of 100 µM GSH, Cys, HCys and various other amino acids (1 – GSH, 2 – Cys, 3 – Hcys, 4 – proline, 5 – serine, 6 – aspartic acid, 7 – lysine, 8 – valine, 9 – isoleucine, 10 – arginine, 11 – histidine, 12 – phenyalanine) in PBS buffer solution, 20 % v/v DMSO, pH 8.00 at 25 ºC. λex = 560 ± 15 nm/ λem = 625 nm

5. Cell viability

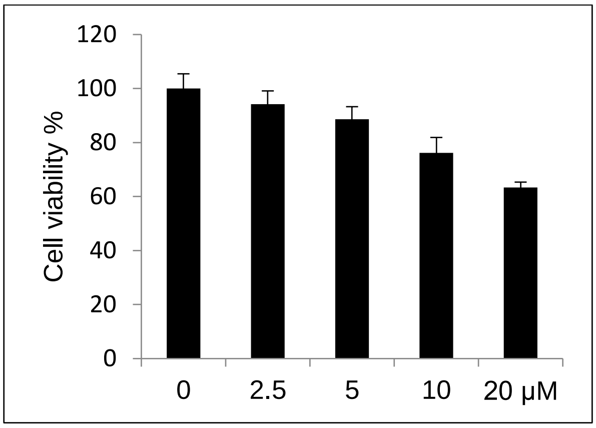

Fig S16. HeLa cells were incubated with each concentration of **TCF-GSH** for 24hr. Cell viability was assayed by MTT test. Results are expressed as mean ± standard deviation of three independent experiments.

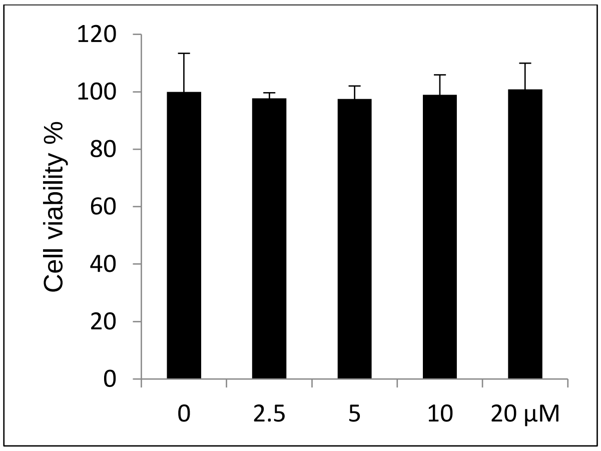

Fig S17. HeLa cells were incubated with each concentration of **TCFCl-GSH** for 24hr. Cell viability was assayed by MTT test. Results are expressed as mean ± standard deviation of three independent experiments.

Method

Cells were seeded in a 96-well plate with culture media. After overnight culture, cells were incubated with each concentration of sample for 24 h. To identify cell viability, reagents were removed and 0.5 mg/ml of MTT (Sigma) media was added to the cells and incubated for 4 hr at 37 ℃CO_2 incubator and the produced formazan was dissolved in 0.1 ml of dimethylsulfoxide (DMSO) and read at OD 650 nm with a Spectramax Microwell plate reader. Absorbance was determined and the mean cell viability was calculated as a percentage of the mean vehicle control and experiments were carried out in three independent tests.

6. Cell imaging

HeLa (human cervix adenocarcinoma) cells were purchased from Korean Cell Line Bank (Seoul, Korea). Cells were cultured in MEM (Eagle's Minimum Essential Medium) supplemented with 10 % fetal bovine serum, 100 U/ml penicillin and 100 U/ml streptomycin and cells were kept in 5 % CO_2 at 37 ℃.

Cells were seeded in a 35-mm glass bottomed dishes at a density of 3 X 10^5 cells per dish in culture media. After overnight culture, HeLa cells were preincubated with 0.2 mM NMM (N-methylmaleimide) for 20 min and washed with DPBS and incubated with 200 μM cysteine, homocysteine and GSH-MEE (cell permeable form of GSH) for 20 min. After washing with DPBS, cells were stained with 20 μM TCF-GSH or TCFCl-GSH for 20 min and acquired fluorescence images by confocal microscopy (FV1200, Olympus). To get the fluorescence image, cells were irradiated with 559 nm laser and used 575-675 nm filter. All experiments were tested in the HBSS (Hanks' Balanced Salt Solution) to prevent another factor interference in the cell culture media.

To reduce intracellular thiol concentrations, cells were treated with 500 μM H_2O_2 or 200 μM cisplatin for 6 hr and 2 mM NAC (N-acetylcysteine) was cotreated to recover this effect.

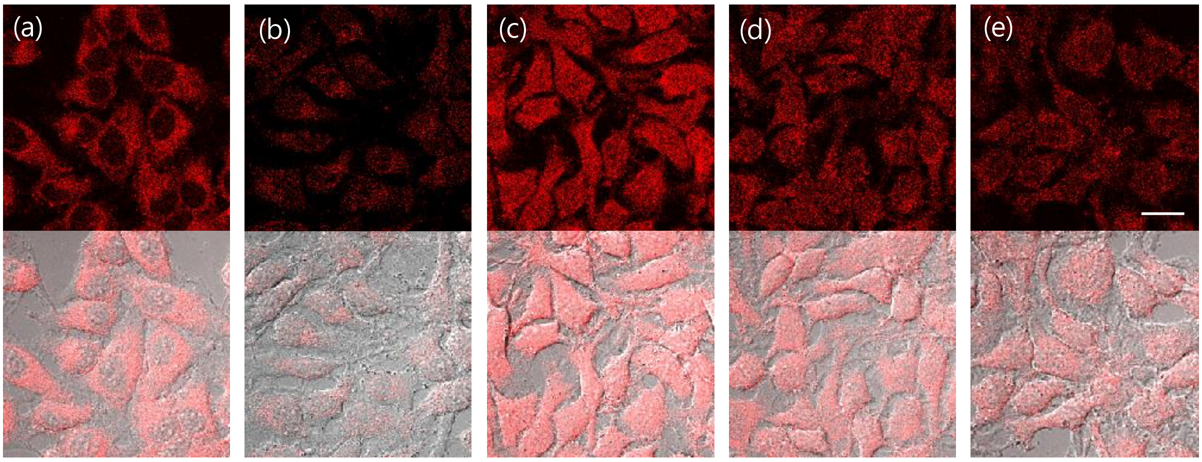

Fig S18. Fluorescence imaging in the live cell. HeLa cells were preincubated with 0.2 mM NMM for 20 min and washed with DPBS and incubated with 200 μM cysteine, homocysteine and GSH-MEE for 20 min. After washing with DPBS, cells were stained with 20 μM TCF-GSH for 20 min and acquired fluorescence images by confocal microscopy. (a) only **TCF-GSH**, (b) NMM + **TCF-GSH**, (c) NMM + cysteine + **TCF-GSH**, (d) NMM + homocysteine + **TCF-GSH** and (e) NMM + GSH-MEE + **TCF-GSH**. Top : fluorescence image (ex. 559 nm/em. 575-675 nm), bottom : merged image with DIC. Scale bar. 20 μm.

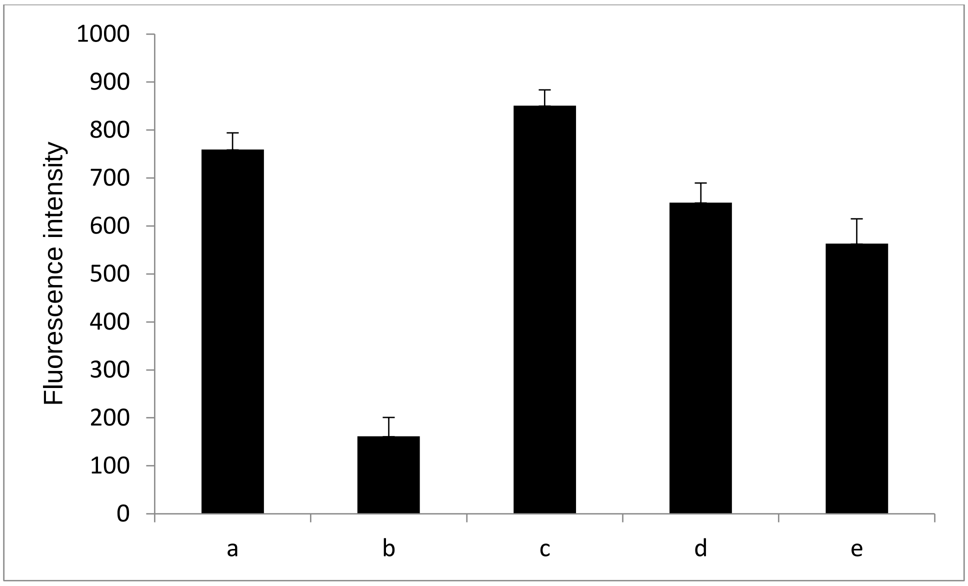

Fig S19. Quantitative data of the above Figure S18. Fluorescence intensity was calculated by FV10-ASW 4.0 software and measured per one cell. Results are expressed as mean ± standard deviation of three independent experiments.

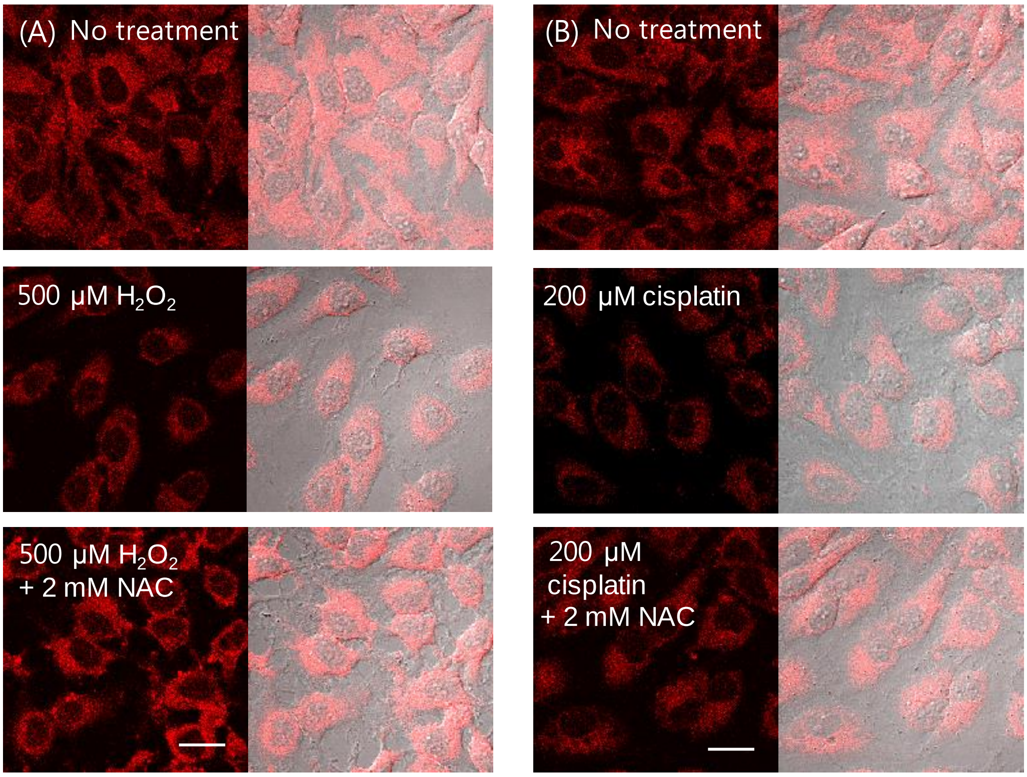

Fig S20. Intracellular fluorescence change by drug treatment. (A) HeLa cells were incubated with 500 µM H_2O_2 with or without 2 mM NAC for 6 hr and stained with 20 µM TCF-GSH for 20 min. (B) HeLa cells were incubated with 200 µM cisplatin with or without 2 mM NAC for 6 hr and stained with 20 µM TCF-GSH for 20 min. Ex. 559 nm/em. 575 – 675 nm. Scale bar : 20 µm.

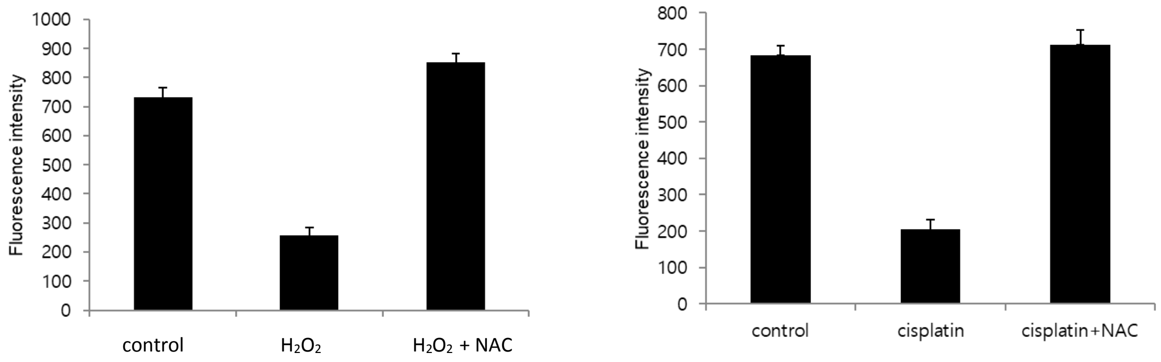

Fig S21. Quantitative data of Fig. S20. Fluorescence intensity was calculated by FV10-ASW 4.0 software and measured per one cell. Results are expressed as mean ± standard deviation of three independent experiments

7. Photobleaching experiments

As shown below in Fig. S22 and S23, photobleaching experiments were carried out against the known biological dye 4’,6-diamidino-2-phenylindole (DAPI). The cells were continuously irradiated and the fluorescence intensity was measured over time. Both TCF-based fluorophores displayed good photostability against DAPI with **TCFCl-GSH** having the best photostability.

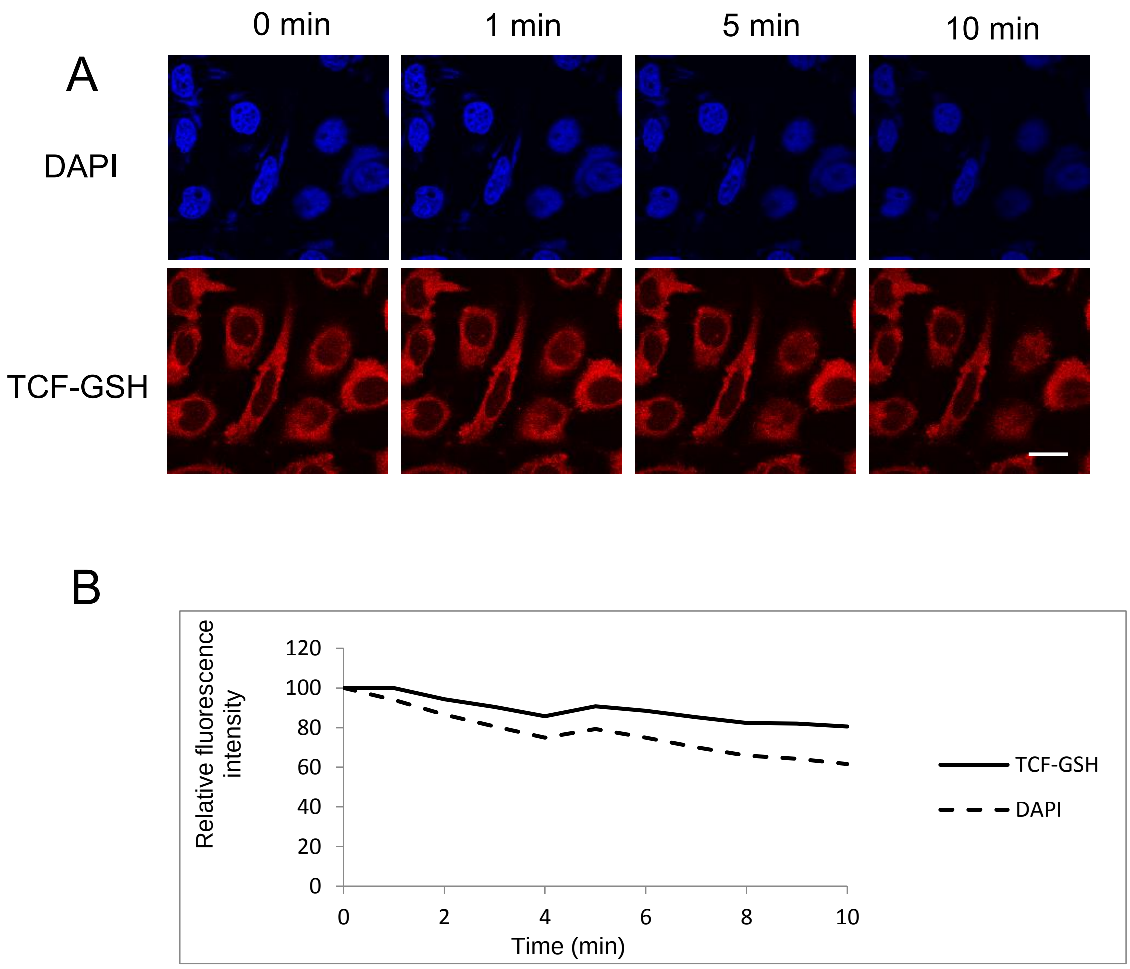

Fig S22. Photobleaching experiments of the **TCF-GSH** in HeLa cells. (A) Cells were costained with **TCF-GSH** and DAPI and acquired fluorescence image by time-dependent manner. Cells were continuously irradiated with laser and the total fluorescence intensity was recorded at every 1 min. Laser power (DAPI : ex. 405 nm/ 50%, **TCF-GSH** : ex. 559 nm/ 50%). Scale bar : 20 µm. (B) Quantitative photobleaching results from (A). Fluorescence Intensity of 0 min was calculated as 100 %.

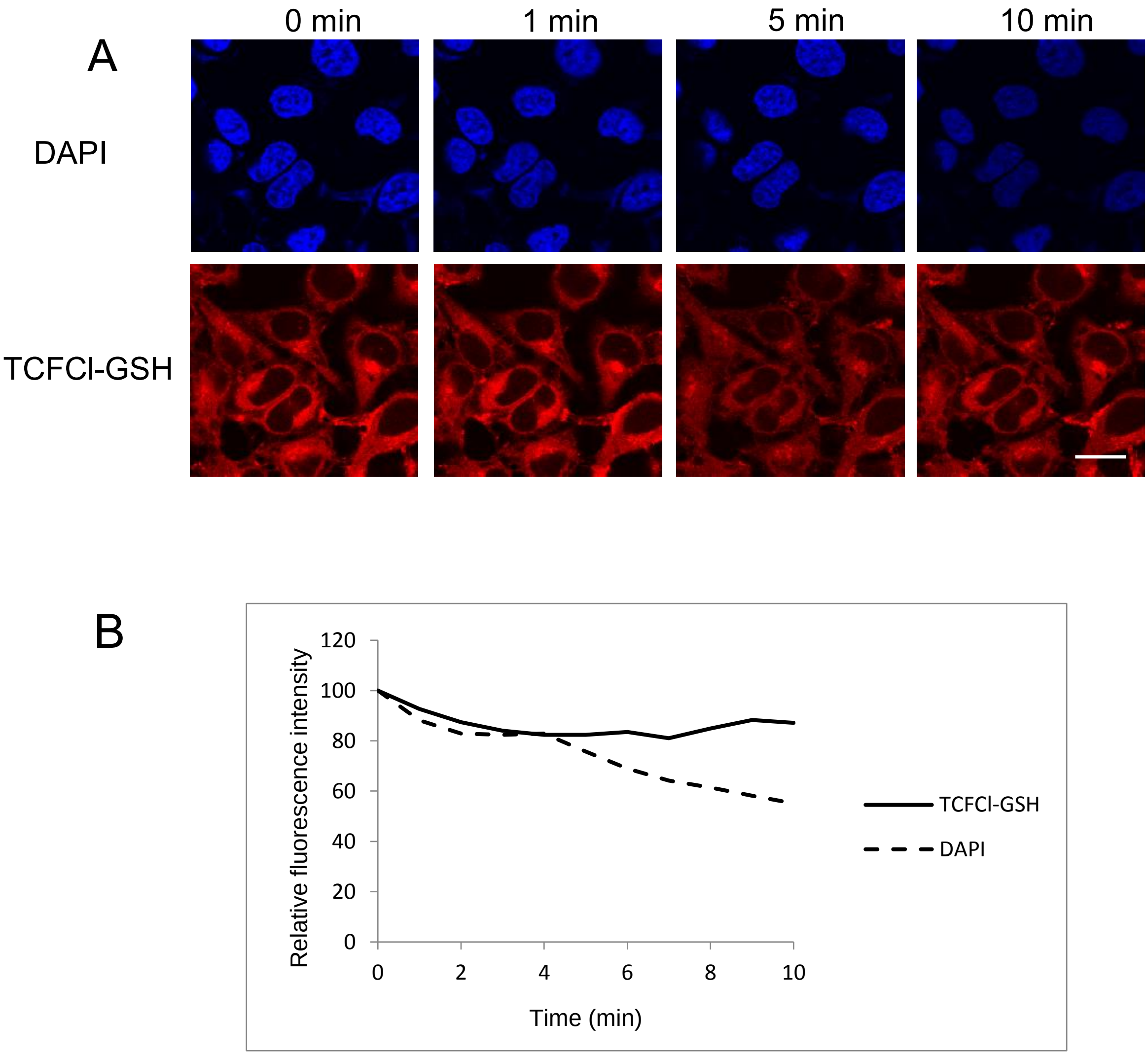

Fig S23. Photobleaching experiments of the **TCFCl-GSH** in HeLa cells. (A) Cells were costained with **TCFCl-GSH** and DAPI and acquired fluorescence image by time-dependent manner. Cells were continuously irradiated with laser and the total fluorescence intensity was recorded at every 1 min. Laser power (DAPI : ex. 405 nm/ 50%, **TCFCl-GSH** : ex. 559 nm/ 50%). Scale bar : 20 µm. (B) Quantitative photobleaching results from (A). Fluorescence Intensity of 0 min was calculated as 100 %.

8. Quantum Yield and Limit of Detection

Table S1 – Quantum Yields for TCF-GSH, TCFCl-GSH, TCF-ClO$^-$ and TCFO$^-$

**Probe**	**Φ**
**TCF-GSH**[(a)]	1.28 x 10^{-4}
**TCFCl-GSH**[(b)]	6.34 x 10^{-4}
**TCF-O**$^{-(c)}$	2 x 10^{-3}
**TCFCl-O**$^{-(c)}$	0.012

(a) The quantum yield of **TCF-GSH** was estimated by dividing **ΦTCFO-** by the 15.63-fold intensity increase for **TCF-GSH** at 615 nm for the addition of GSH (63 µM). (b) The quantum yield of **TCFCl-GSH** was estimated by dividing **ΦTCFCl-O**$^-$ by the 18.94-fold intensity increase for **TCFCl-GSH** at 625 nm for the addition of GSH (750 µM). (c) The quantum yield of **TCFO-** (**ΦTCFO-)** and **TCFClO-** (**ΦTCFClO-)** were previously reported.[2]

Limit of detection

The limit of detection was calculated using the formula shown below:

Limit of detection (LOD) = 3σ/slope

σ = Standard deviation of the lowest concentration

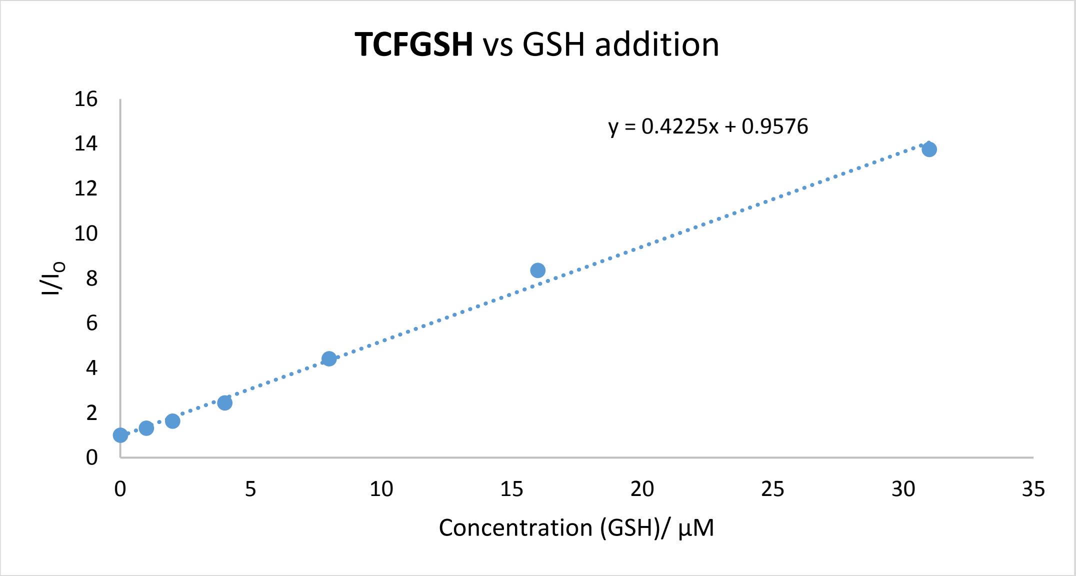

Fig S24. Fluorescence intensity changes of **TCF-GSH** with the addition of GSH) in PBS buffer solution, 20 % DMSO, pH 8.00 at 25 ºC. λex = 560 ± 15 nm/ λem = 615 nm

LOD for **TCF-GSH** = (3 x 0.0396)/0.4225 = 0.28 µM

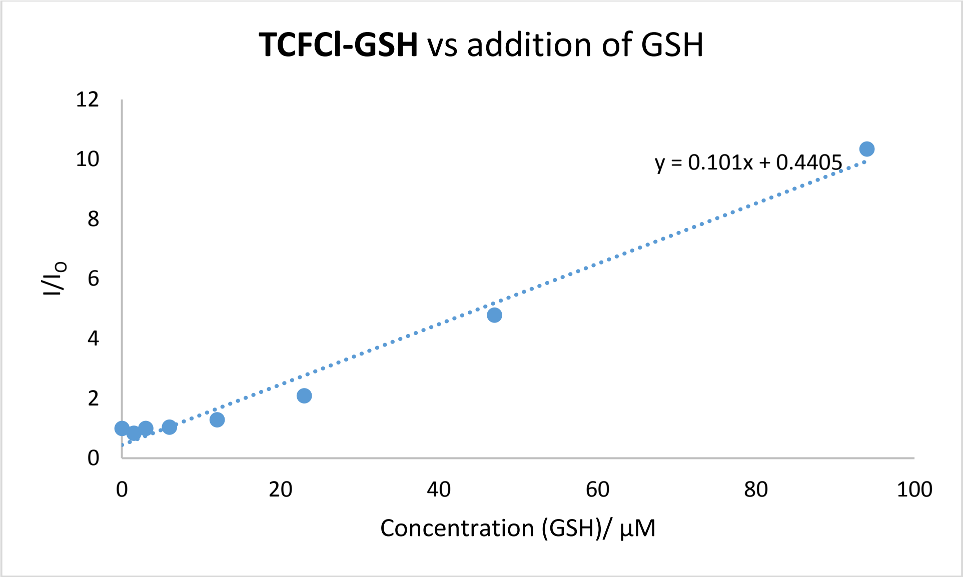

Fig S25. Fluorescence intensity changes of **TCFCl-GSH** with the addition of GSH in PBS buffer solution, 20 % v/v DMSO, pH 8.00 at 25 ºC. λex = 560 ± 15 nm/ λem = 625 nm

LOD for **TCFCl-GSH** = (3 x 0.0153)/0.101 = 0.45 µM

9. Experimental

2-(3-cyano-4,5,5-trimethylfuran-2(5H)-ylidene)malononitrile (1)

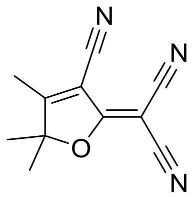

NaOEt (0.391 g, 5.75 mmol) was added to a solution of 3-hydroxy-3-methyl-2-butanone (4 mL, 38 mmol) and malonitrile (4.9 g, 74 mmol) in EtOH (10 mL) and stirred for 1.5 h. The reaction mixture was then refluxed for 1 h, which was then cooled to rt. The mixture was cooled and the solid precipitate was filtered to afford the title compound as a pale grey solid (4.92 g, 24.70 mmol, 65 %); M.p. 204 – 208 °C (decomp). ^{1}H NMR (500 MHz, $CDCl_3$) δ 2.37 (s, 3 H), 1.64 (s, 6 H); ^{13}C NMR (75.5 MHz, $CDCl_3$) δ 182.6, 175.2, 111.1, 110.4, 109.0, 104.8, 99.8, 58.5, 24.4, 14.2; I.R (thinfilm) ν max (cm^{-1}): 2232.78, 2222.00 (CN); HRMS (FTMS-NSI): m/z calculated for $C_{11}H_9N_3O$: requires 200.0108 for $[M+H]^+$, found 200.0108.

(*E*)-2-(3-cyano-4-(4-hydroxystyryl)-5,5-dimethylfuran-2(5H)-ylidene)malononitrile (2)

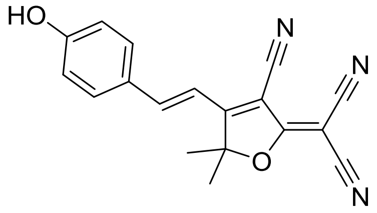

Two drops of piperidine were added to a mixture of 4-hydroxybenzaldehyde (0.122 g, 1 mmol) and TCF (0.228 g, 1.15 mmol) in EtOH (10 mL). The reaction mixture was heated in the microwave for 15 min at 100 °C, which was then cooled to rt. The solid precipitate was filtered off to afford the title compound as an orange solid (0.218 g, 0.72 mmol, 72 %) M.p. 202 – 206 °C (decomp). ^{1}H NMR (300MHz, DMSO-d_6) 7.95 - 7.73 (m, 3 H), 7.01 (d, J = 16.2 Hz, 1 H), 6.89 (d, J = 8.7 Hz, 2 H), 1.77 (s, 6 H); ^{13}C NMR (75.5 MHz, DMSO-d_6) δ 177.6, 176.2, 162.7, 148.7, 132.7, 126.0, 116.8, 113.3, 112.5, 112.0, 111.6, 99.4, 96.9, 53.5, 25.7; I.R (thinfilm) ν max (cm^{-1}): 3361.61 (O-H), 2224.73 (CN); HRMS (FTMS-NSI): m/z calculated for $C_{18}H_{13}N_3O_2$: requires 304.1081 for $[M+H]^+$, found 304.1084.

(*E*)-4-(2-(4-cyano-5-(dicyanomethylene)-2,2-dimethyl-2,5-dihydrofuran-3yl)vinyl)phenyl 2,4-dinitrobenzenesulfonate (3)

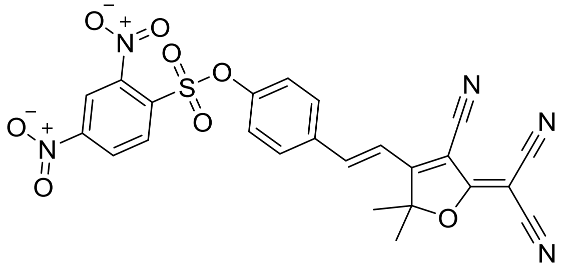

2 (0.10 g, 0.33 mmol) was dissolved in DCM (5 mL) followed by the addition of NEt_3 (89 µL, 0.66 mmol). The reaction mixture was cooled to 0 °C and 2,4-dinitrobenzenesulfonylchloride (0.11 g, 0.40 mmol) in DCM (5 mL) was added dropwise to the reaction mixture. The reaction was allowed to warm to rt and was stirred for a further 4 hrs. The solvent was removed *in vacuo* and the crude product was purified *via* column chromatography - EtOAc:Pet ether (20;:80 to 60:40). The title compound was isolated as an orange solid (0.08 g, 0.15 mmol, 45 %). M.p . – 236 – 238 °C; ^{1}H NMR (300 MHz, DMSO-d_6) δ 9.13 (d, *J* = 2.3 Hz, 1 H), 8.59 (dd, *J* = 2.3, 8.7 Hz, 1 H), 8.24 (d, *J* = 8.7 Hz, 1 H), 7.99 (d, *J* = 8.9 Hz, 2 H), 7.87 (d, *J* = 16.8 Hz, 1 H), 7.32 (d, *J* = 8.9 Hz, 2 H), 7.21 (d, *J* = 16.6 Hz, 1 H), 1.77 (s, 6 H); ^{13}C NMR (125. 75 MHz, Acetone-d6) δ 176.3, 174.4, 151.0, 144.7, 134.7, 134.0, 131.9, 131.1, 127.2, 123.0, 120.9, 117.0, 111.9, 111.2, 110.2, 101.4, 99.1, 25.0; I.R (thinfilm) ν max (cm^{-1}): 3100 (C-H sp^2), 2229.72 (CN); HRMS (FTMS-NSI): m/z calculated for $C_{24}H_{15}N_5O_8S$: requires 534.0714 for $[M+H]^+$, found 534.0720

(*E*)-2-(3-cyano-4-(3,5-dichloro-4-hydroxystyryl)-5,5-dimethylfuran-2(5H)-ylidene)malononitrile (4)

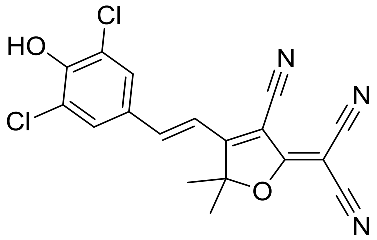

Two drops of piperidine were added to a mixture of 3,5-dichloro-4-hydroxybenzaldehyde (0.19 g, 1 mmol) and TCF (0.20 g, 1.00 mmol) in EtOH (10 mL). The reaction mixture was heated in the microwave for 15 min at 100 ^oC, which was then cooled to rt. The solid precipitate was filtered off to afford the title compound as a blue solid (0.19 g, 0.50 mmol, 50 %). M.p. - > 300 ^oC; ^1H NMR (500 MHz, DMSO-d6) δ 8.06 (s, 2 H), 7.76 (d, J = 16.1 Hz, 1 H), 7.19 (d, J = 16.1 Hz, 1 H), 1.77 (s, 6 H); ^{13}C NMR (125.75 MHz, DMSO-d6) δ 177.5, 175.4, 153.2, 145.4, 130.4, 127.6, 123.3, 115.1, 113.2, 112.3, 111.2, 99.7, 25.6; I.R (thinfilm) ν max (cm^{-1}): 3356.50 (Br O-H), 2212.91 (CN); HRMS (ASAP+): m/z calculated for $C_{18}H_{11}Cl_2N_3O_2$: requires 372.0307 for $[M+H]^+$, found 372.0300

(*E*)-2,6-dichloro-4-(2-(4-cyano-5-(dicyanomethylene)-2,2-dimethyl-2,5-dihydrofuran-3-yl)vinyl)phenyl 2,4-dinitrobenzenesulfonate (5)

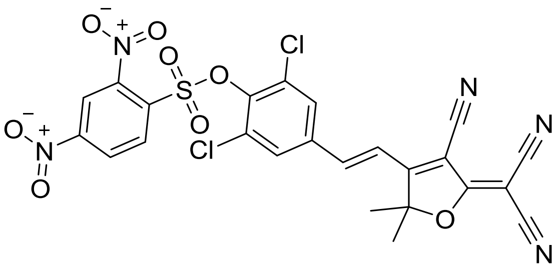

4 (0.17 g, 0.46 mmol) was dissolved in DCM (5 mL) followed by the addition of NEt_3 (124 µL, 0.92 mmol). The reaction mixture was cooled to 0 °C and 2,4-dinitrobenzenesulfonylchloride (0.146 g, 0.55 mmol) in DCM (5 mL) was added dropwise to the reaction mixture. The reaction was allowed to warm to rt and was stirred for a further 4 hrs. The solvent was removed *in vacuo* and the crude product was purified *via* column chromatography - EtOAc:Pet ether (60:40). The title compound was isolated as a yellow solid (0.20 g, 0.33 mmol, 72 %). M.P. – 268 -272 °C; ^{1}H NMR (500 MHz, DMSO-d6) δ 9.12 (d, *J* = 2.4 Hz, 1 H), 8.70 (dd, *J* = 2.4, 8.8 Hz, 1 H), 8.49 (d, *J* = 8.8 Hz, 1 H), 8.34 (s, 2 H), 7.76 (d, *J* = 16.6 Hz, 1 H), 7.44 (d, *J* = 16.6 Hz, 1 H), 1.80 (s, 6 H); ^{13}C NMR (125.75 MHz, DMSO-d6) δ 177.3, 174.2, 152.1, 148.2, 143.7, 142.6, 136.5, 133.6, 133.4, 130.5, 129.9, 128.3, 121.6, 119.7, 112.9, 112.1, 110.7, 102.4, 100.1, 56.0, 25.4; I.R (thinfilm) ν max (cm^{-1}): 3112.22 (C-H sp^2), 2225.72 (CN); HRMS (FTMS-NSI): m/z calculated for $C_{24}H_{13}Cl_2N_5O_8S$: requires 623.9754 for [M+Na]$^+$, found 623.9761

10. NMR

2-(3-cyano-4,5,5-trimethylfuran-2(5H)-ylidene)malononitrile (1) (500 MHz, $CDCl_3$)

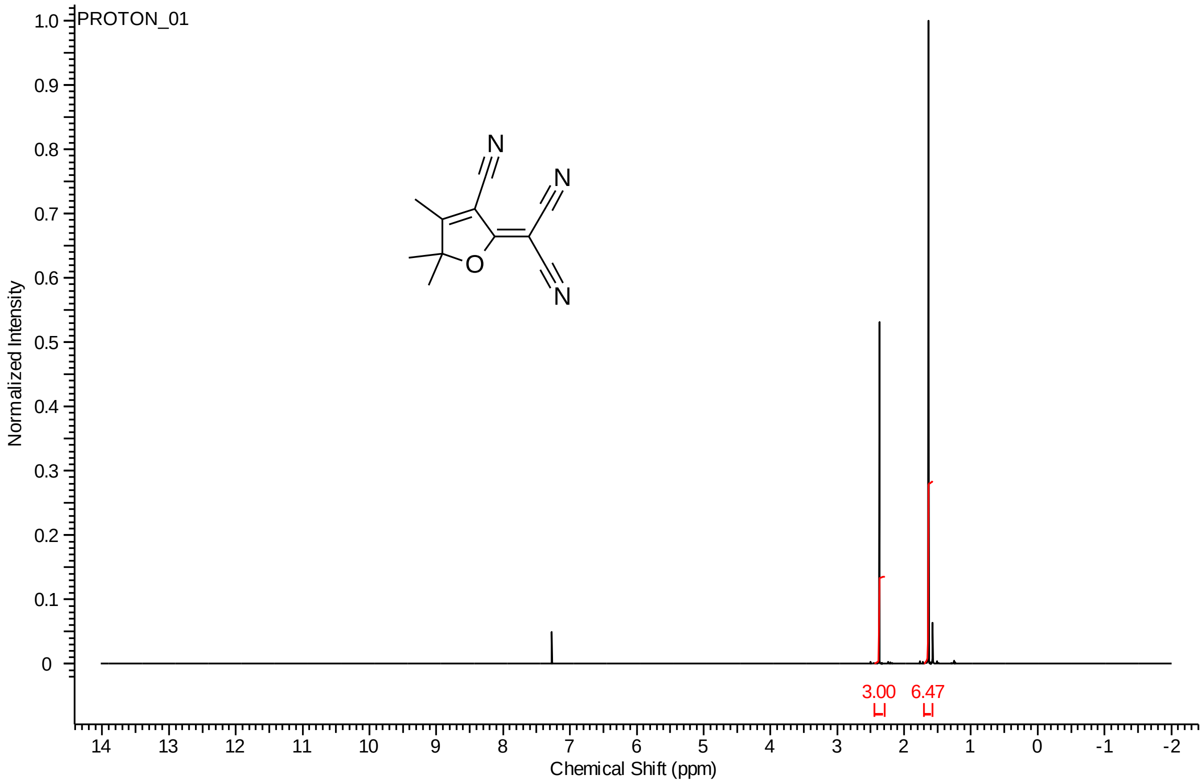

2-(3-cyano-4,5,5-trimethylfuran-2(5H)-ylidene)malononitrile (1) (75.5 MHz, $CDCl_3$)

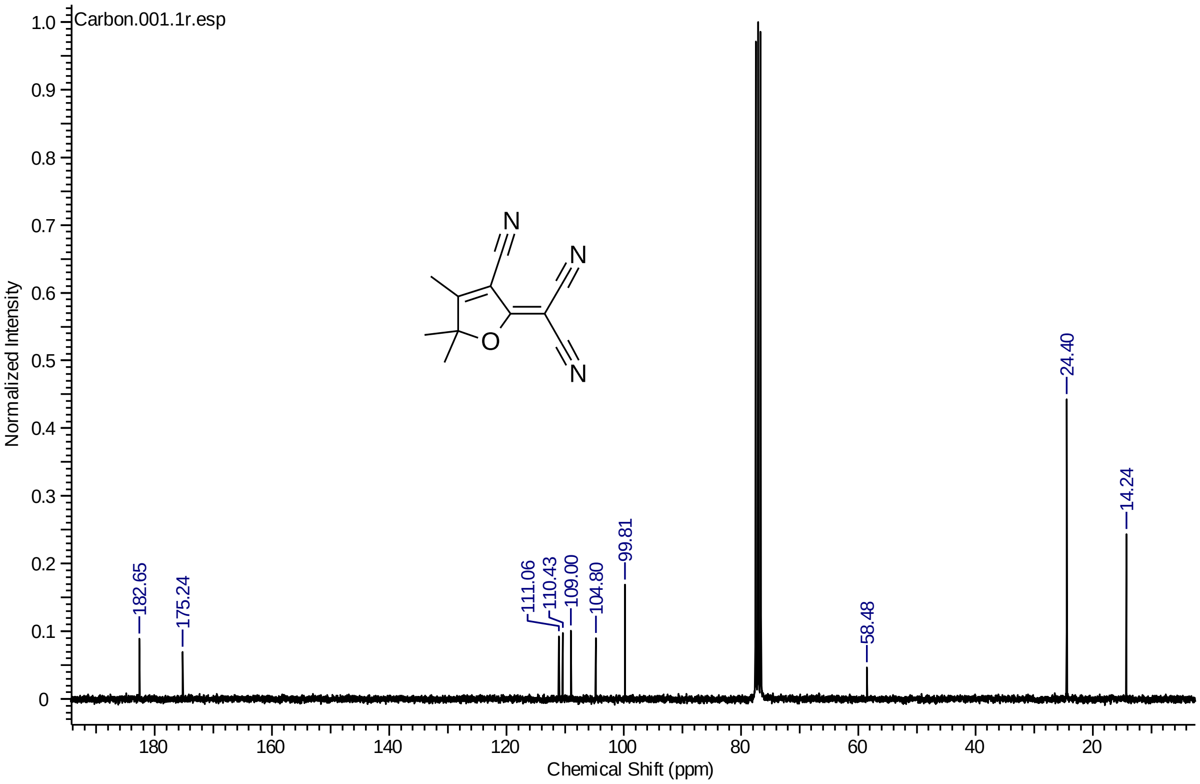

(E)-2-(3-cyano-4-(4-hydroxystyryl)-5,5-dimethylfuran-2(5H)-ylidene)malononitrile (2)
(300 MHz, $CDCl_3$)

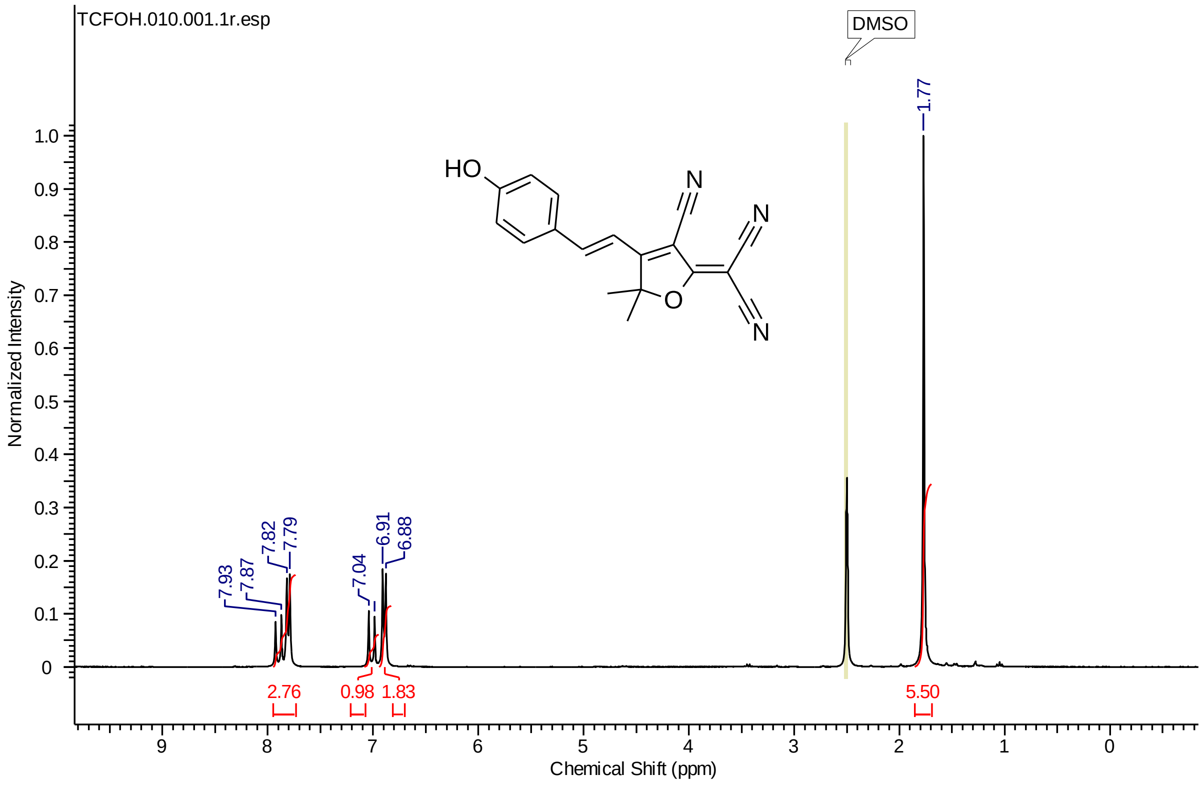

(E)-2-(3-cyano-4-(4-hydroxystyryl)-5,5-dimethylfuran-2(5H)-ylidene)malononitrile (2)
(75.5 MHz, $CDCl_3$)

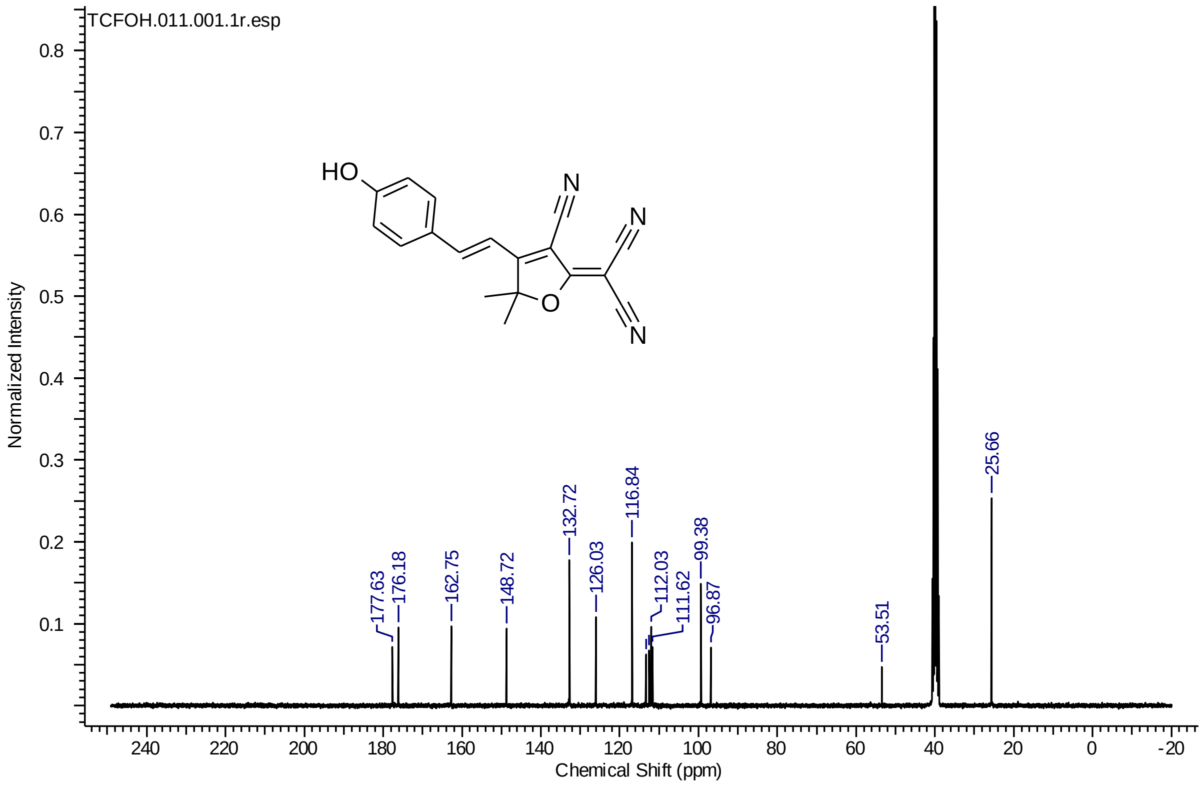

(*E*)-4-(2-(4-cyano-5-(dicyanomethylene)-2,2-dimethyl-2,5-dihydrofuran-3yl)vinyl)phenyl 2,4-dinitrobenzenesulfonate (3) (300 MHz, DMSO-d6)

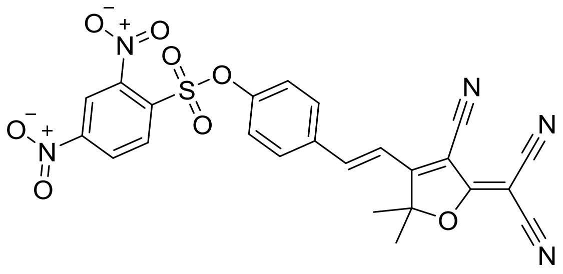

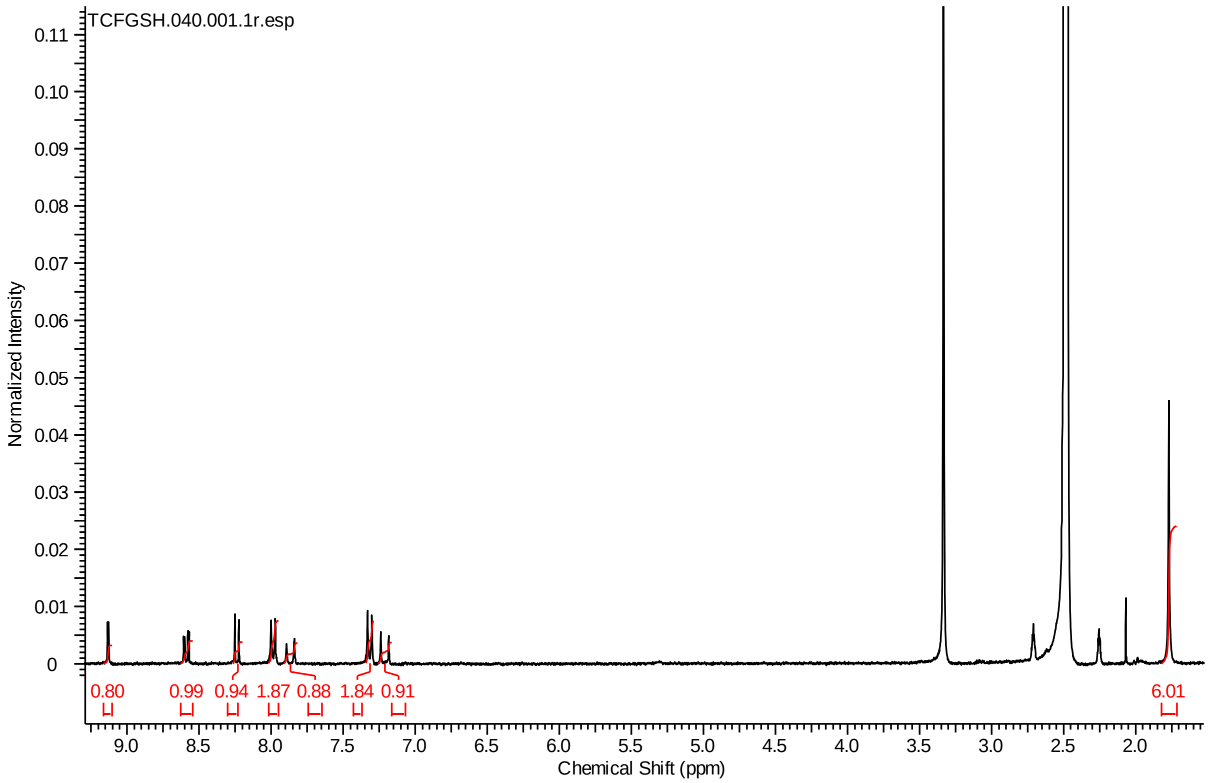

(*E*)-4-(2-(4-cyano-5-(dicyanomethylene)-2,2-dimethyl-2,5-dihydrofuran-3yl)vinyl)phenyl 2,4-dinitrobenzenesulfonate (3) (125.75 MHz, DMSO-d6)

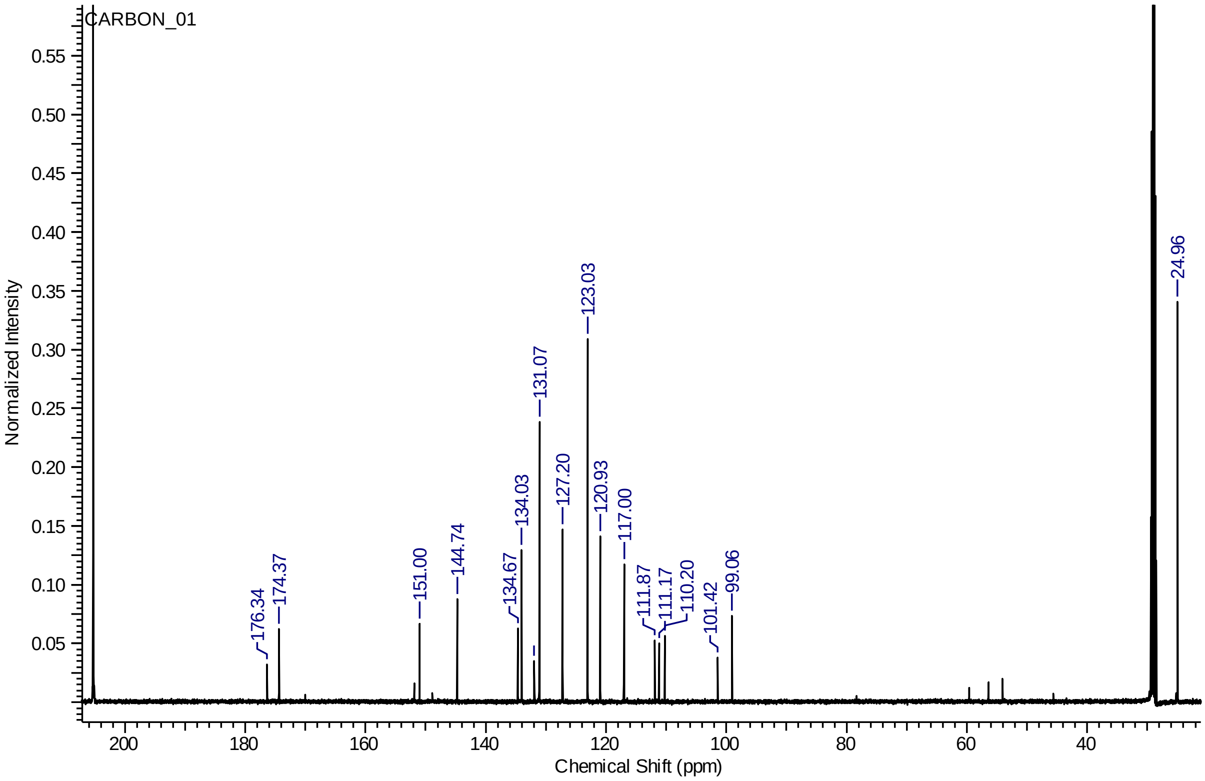

(*E*)-2-(3-cyano-4-(3,5-dichloro-4-hydroxystyryl)-5,5-dimethylfuran-2(5H) ylidene)malononitrile (4) (500 MHz, DMSO-d6)

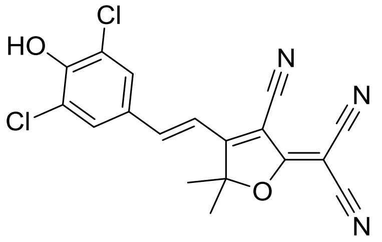

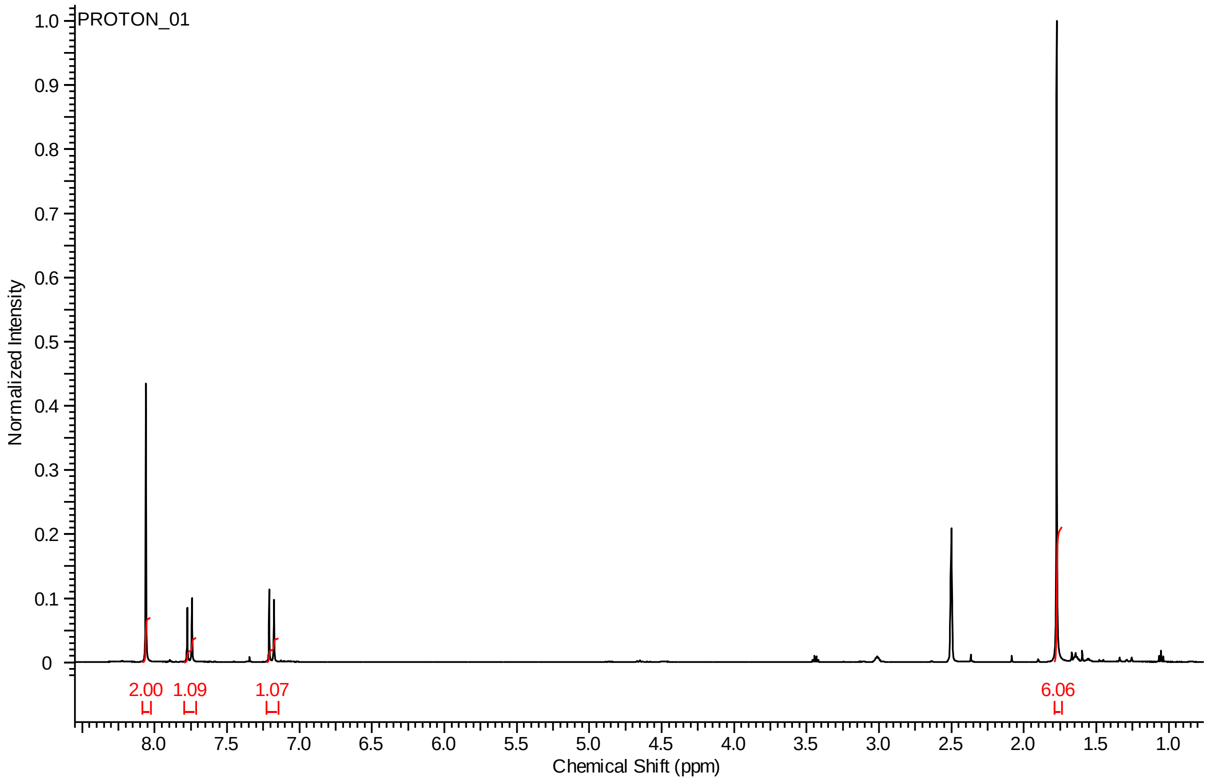

(*E*)-2-(3-cyano-4-(3,5-dichloro-4-hydroxystyryl)-5,5-dimethylfuran-2(5H) ylidene)malononitrile (4) (125.75 MHz, DMSO-d6)

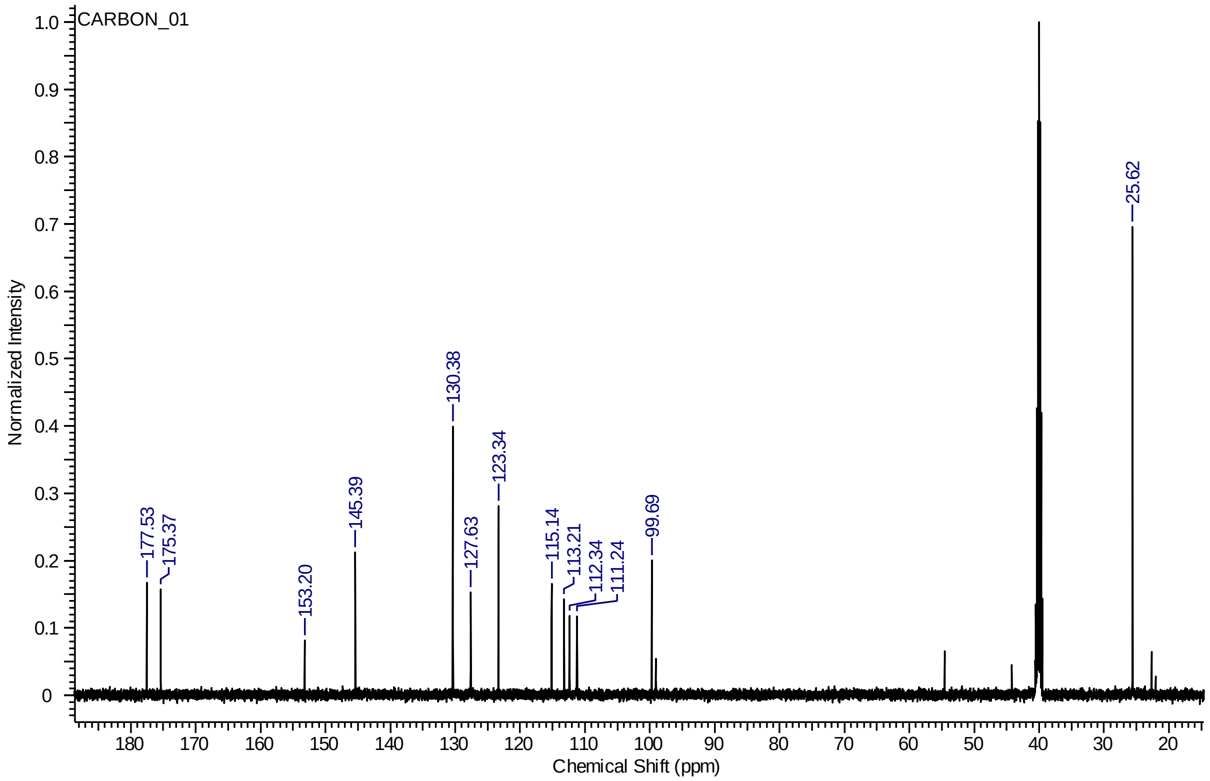

(*E*)-2,6-dichloro-4-(2-(4-cyano-5-(dicyanomethylene)-2,2-dimethyl-2,5-dihydrofuran-3-yl)vinyl)phenyl 2,4-dinitrobenzenesulfonate (5) (500 MHz, DMSO-d6)

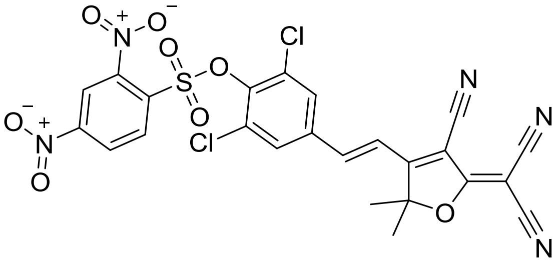

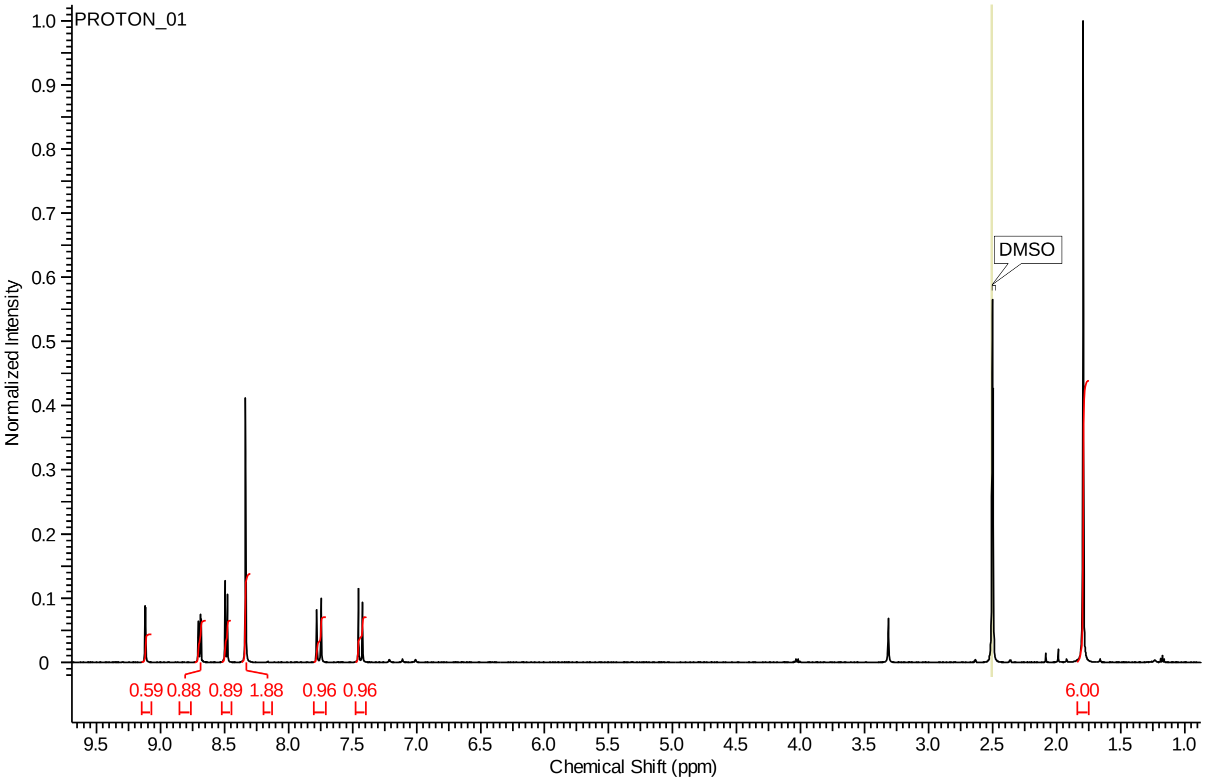

(*E*)-2,6-dichloro-4-(2-(4-cyano-5-(dicyanomethylene)-2,2-dimethyl-2,5-dihydrofuran-3-yl)vinyl)phenyl 2,4-dinitrobenzenesulfonate (5) (125.75 MHz, DMSO-d6)

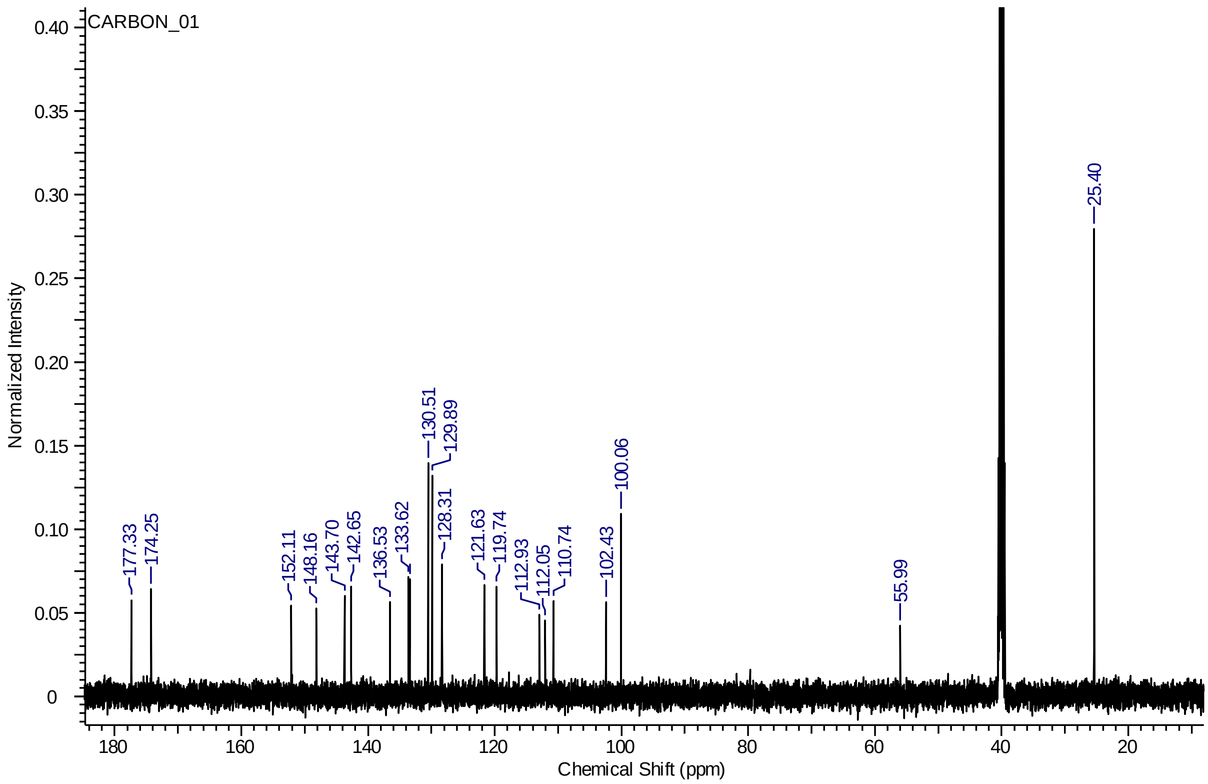

11. References

(1) Zhu, B. C.; Kan, H.; Liu, J. K.; Liu, H. G.; Wei, Q.; Du, B. A highly selective ratiometric visual and red-emitting fluorescent dual-channel probe for imaging fluoride anions in living cells. *Biosens. Bioelectron.* **2014,** *52*, 298.

(2) Ipuy, M.; Billon, C.; Micouin, G.; Samarut, J.; Andraud, C.; Bretonniere, Y. Fluorescent push-pull pH-responsive probes for ratiometric detection of intracellular pH. *Org. Biomol. Chem.* **2014,** *12* (22), 3641.
